# Humans depart from optimal computational models of interactive decision-making during competition under partial information

**DOI:** 10.1038/s41598-021-04272-x

**Published:** 2022-01-07

**Authors:** Saurabh Steixner-Kumar, Tessa Rusch, Prashant Doshi, Michael Spezio, Jan Gläscher

**Affiliations:** 1 Institute of Systems Neuroscience, University Medical Center Hamburg-Eppendorf, Hamburg, Germany; 2 Division of the Humanities and Social Sciences, California Institute of Technology, Pasadena, CA USA; 3 Department of Computer Science, University of Georgia, Athens, GA USA; 4 Psychology, Neuroscience, and Data Science, Scripps College, Claremont, CA USA

**Keywords:** Human behaviour, Cooperation, Reward, Social behaviour, Decision

## Abstract

Decision making under uncertainty in multiagent settings is of increasing interest in decision science. The degree to which human agents depart from computationally optimal solutions in socially interactive settings is generally unknown. Such understanding provides insight into how social contexts affect human interaction and the underlying contributions of Theory of Mind. In this paper, we adapt the well-known ‘Tiger Problem’ from artificial-agent research to human participants in solo and interactive settings. Compared to computationally optimal solutions, participants gathered less information before outcome-related decisions when competing than cooperating with others. These departures from optimality were not haphazard but showed evidence of improved performance through learning. Costly errors emerged under conditions of competition, yielding both lower rates of rewarding actions and accuracy in predicting others. Taken together, this work provides a novel approach and insights into studying human social interaction when shared information is partial.

## Introduction

Formal computational models of agent actions can identify optimal sequences of exploration and/or option selection. Ideally, these models will allow for robust artificial intelligence (AI) systems that can pair with human agents in contexts of competition and cooperation. These contexts include socially assistive robotics^1–3^ and machine assisted cognition or decision making^4–7^. To achieve this goal, principled design for machine-assisted cognition must move beyond modeling normative actions and action probabilities, and develop accurate models of the beliefs and intentions of the human agents^8^. Following such a research program gives the best chance of yielding useful adaptations of machine-based guidance in shifting contexts. Such models require accounting for how human agents’ beliefs, intentions, and actions differ from computationally optimal solutions, since what is computationally optimal is rational often under the conditions of narrow, static value functions and a limited array of learning algorithms. One especially needs to know how these differences are affected by changes in the competitive and cooperative environments which in turn influence human state representation and valuation of gains and losses for self^9,10^ and others^11^. Progress in meeting these requirements will involve studying how human agents act under uncertainty in the same simulated partially observable tasks that are also used to advance robotic and other computational agents in multiagent interactions.

Several social neuroscience studies have similarly embraced a Bayes-optimal approach to modeling social influence in competitive and cooperative choices in human participants^12–15^, while others have argued that social norms like reciprocity^16^, an equality bias^17^, or even their own previous choices^18^ affects human decision-making, which therefore departs from optimal Bayesian integration of social information. In this paper we follow-up on this distinction and ask the question if and how the interactional context of cooperation and competition under partial observability affects their abilities to perform comparatively to a Bayes-optimal decision strategy.

The fields of operations research and of artificial intelligence in computer science have long investigated challenges of this kind^19–21^. In a seminal paper, Kaelbling and colleagues^22^ introduced a novel simulation problem environment (the Tiger Problem) and a model for investigating decisions in which an agent needs to develop beneficial strategies or policies thereby maximizing its *expected reward*^9,23,24^, when they only have partial information about the state of the environment.

One of the limitations of the original Tiger Problem is that it was incapable of addressing multiagent contexts in which agents must develop models of and be sensitive to other agents’ representations of the environment. These contexts require some way of formally modeling the models of other agents, that is, modeling a “theory of mind” (ToM^25^), especially in situations where both the environment and other agents’ actions constitute critical uncertainties in decision making. To overcome this limitation we introduced the interactive Tiger Problem (ITP), along with a modeling solution^26^.

We adapted the Tiger Problem from AI^22,26^ to the domains of human action and interaction, under both cooperative and competitive contexts^27^ and developed a single-agent Tiger Task (TT) and a dyadic Interactive Tiger Task (ITT) respectively. In both versions of the task the participant has to choose between two doors hiding a small reward or a larger punishment (the tiger). They can gather evidence through a number of Listen actions that give rise to probabilistic information about the tiger’s location. In the ITT the participants also receive probabilistic information about the likely action of the other player. The joint payout matrix for both players induces different interactional context: during cooperation the highest rewards are obtained for jointly opening the correct door, whereas during competition the highest reward is obtained when opening the correct door before the other player.

Using modifications of the payout matrices originally developed by Doshi^27^, we compared human choices in the TT and in both the contexts of the ITT to computationally optimal solutions of the ITP. In our ITT design, we also added a novel aspect which required participants to predict the action of the other agent prior to choosing their own action. This task design feature increases the likelihood that participants focus explicitly on the other person and their actions, which increases the likelihood of participants’ engaging Theory of Mind (ToM) processing^28,29^. This step should elicit data that allows us to model and assess putative ToM processes more directly in our future work.

In this article, we sought to identify if and how varying contexts influence departures from theoretical optima, and to compare them to the departures in the TT. Further, we assessed whether these departures might benefit human agents by associating with better actions and predictions in reaching the reward. Machine assisted cognition will fail in its goals, if its models fail to capture what real human agents actually value, believe, and do. Computational optimality will in fact become suboptimal, if the recommended interactive options result only from static, artificial value functions and do not adapt to human agents’ departures from those functions. In complex multiagent interactions for which human agents have some evolved expertise, human situated cognition may yield values, beliefs, and actions that end in more optimal outcomes than those driven by limited value functions and learning algorithms, especially under conditions of limited data availability. Even in a simplified task context such as the ITT, we would expect that human agents can produce accurate predictions of other agents’ actions, when they model others’ beliefs and expected values.

We tested and report two versions of the TT and ITT defined by algorithms for constructing payout matrices. In the first set of payout matrices introduced by Kaelbling et al.^22^ and Gmyntrasiewicz and Doshi^26^, the expected values of two Open actions differed starkly because of the large losses incurred when opening the tiger door. Here, we expected longer evidence gathering by our participants in order to construct precise belief estimates for both doors. In contrast, in our second set of payout matrices the losses after opening the tiger door are halved (more forgiving of errors), which we expected would result in fewer Listen action (less evidence gathering).

In the ITTs, we expected that varying interactional contexts would lead to divergent response patterns. Under competition, we expected that people would race the other participant to the correct decision, and so would gather less evidence than in the TT, despite the ITT’s requiring additional cognitive load to integrate partial observations of others’ actions. Conversely, we expected cooperation to elicit greater care and coordination resulting in longer evidence gathering so that both participants would have the best opportunities to maximize their joint rewards. Since better evidence should yield improved estimates of others’ models and actions, we expected cooperation to yield more accurate predictions about other agents’ actions. Also, given that cooperation incentivizes careful coordination, we expected that people would choose actions consistent with their predictions more often during cooperation. This expectation is consistent with greater explicit or implicit confidence in the evidence underlying those predictions or in the predictions themselves.

## Results

**Figure 1:**
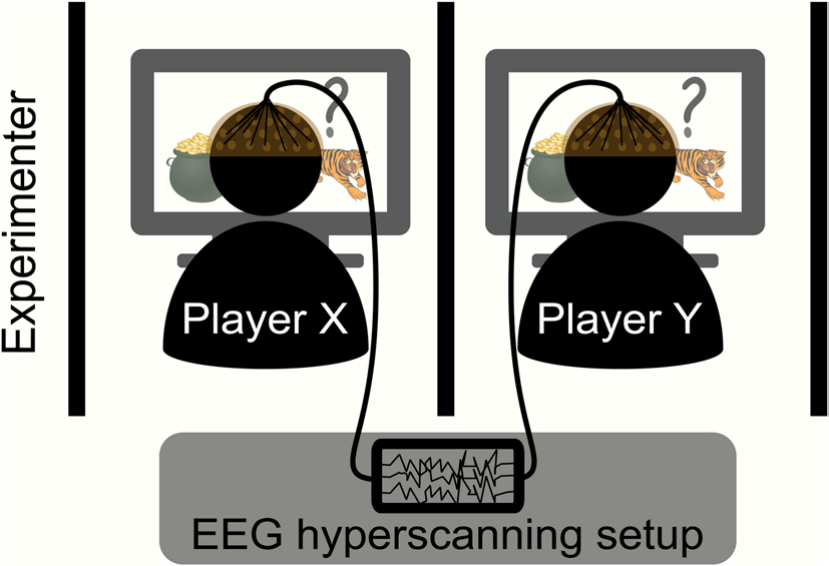
Task schematic of the experiment. The dyad (two participants X and Y) were separated by a partition. Each participant faced their own computer screen playing the tiger task and wore headphones playing constant static to ensure that they could not hear key presses or other low level sounds. Additionally, each wore an EEG cap in a hyperscanning setup. The experimenter silently monitored the behavior and EEG from behind another partition.

**Figure 2:**
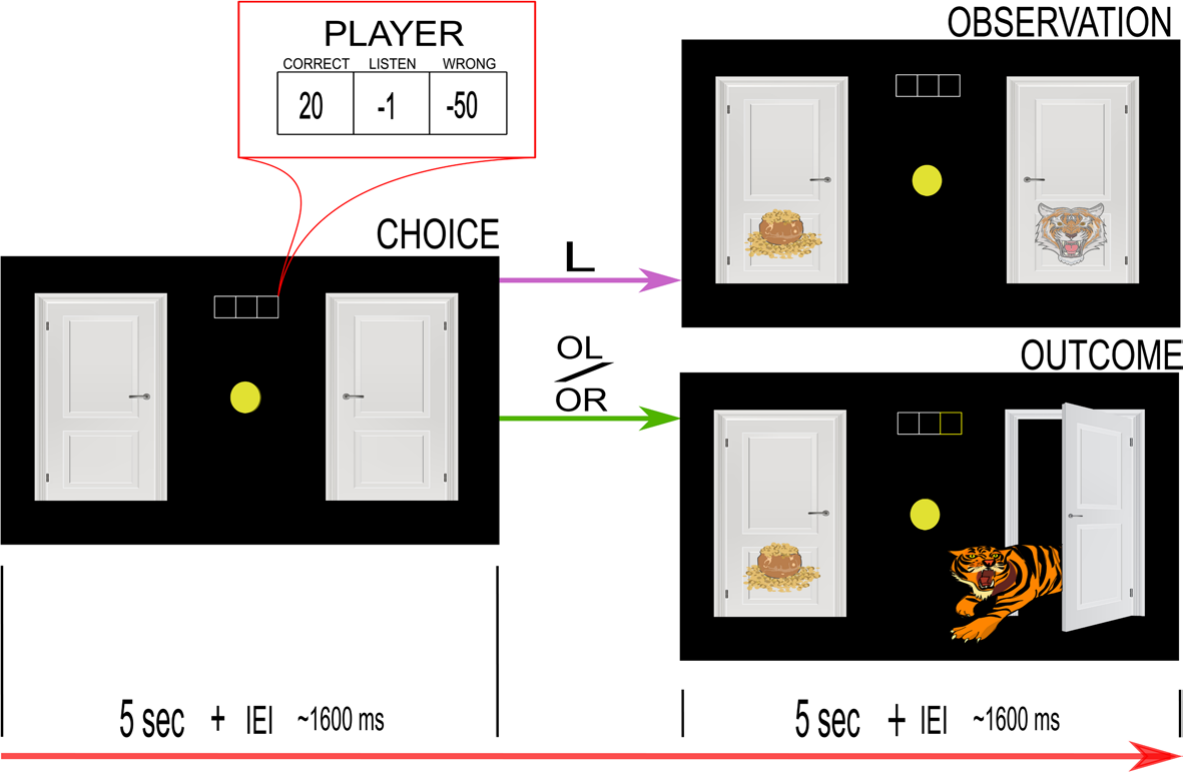
Task sequence of the single-agent version of the tiger task (TT). At the beginning of each decision step, the participant saw two doors and made an action choice (CHOICE) from a set of three actions (Listen (L), Open-left (OL), Open-right (OR)). Participants had 5 seconds to make their choice, else a default listen action was chosen for them. A chosen listen action led to a probabilistic observation (PHYSICAL-OBSERVATION, $70\%$ correct), and the sequence repeated. The probabilistic hints were signified by semi-transparent images of the tiger and the gold-pot on the respective doors. There was always an inter-event-interval (IEI) of $\sim 1600$ milliseconds. Choosing left/right open action opened the door (OUTCOME), where the participants either received the gold-pot or encountered the tiger (tiger encounter is shown here). Opening the door reset the tiger and the reward to begin the task sequence again. The matrix in the red box shows the potential payouts the participant could expect upon the different action choices (constantly displayed through the TT).

**Figure 3:**
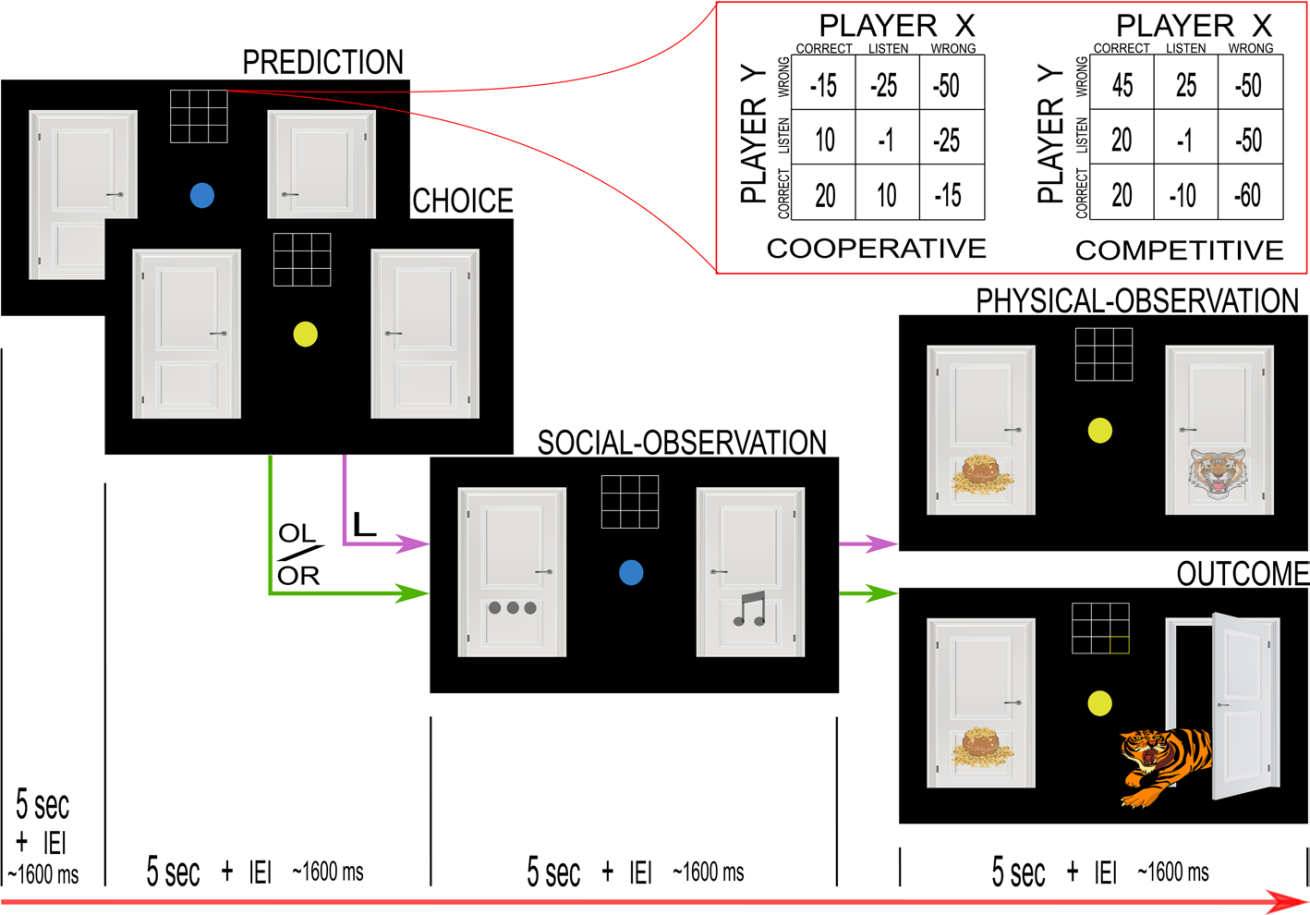
Task sequence of the multiagent version (cooperative and competitive context) of the interactive tiger task (ITT). The task sequence was color-coded for the participant. Blue represented the other participant and yellow represented self. The participant began each round of the task by predicting the action choice of the other participant (PREDICTION). The participant had 5 seconds. After an inter-event-interval (IEI) of $\sim 1600$ milliseconds, the participant had to make the personal action choice (CHOICE) within 5 s. Regardless of the one’s own action, the participant obtained probabilistic ($80\%$ correct) social information (SOCIAL-OBSERVATION) about the other participant before either receiving the probabilistic hint ($70\%$ correct) about the locations of the tiger and the pot of gold (PHYSICAL-OBSERVATION) for Listen (L) action, or opening the door (OUTCOME) for open left/right (OL/OR) actions, to get the gold-pot/tiger (tiger encounter is shown in the figure). Opening the door randomly reallocated the tiger and the reward locations to begin the tiger-trial sequence again. The permanent context-specific matrices in the red box show the potential payouts for the participant (player X) during interaction with the other (player Y).

In the task, individual trials began with two doors presented on the computer screen. Participants understood that each door concealed either a tiger or a pot of gold (i.e., reward). The task was to open the correct door (i.e., the one hiding the pot of gold) and avoid the tiger. On each round, a participant had a set of three different actions available: listen (L), open the door on the left (OL), and open the door on the right (OR). The L action gave a probabilistic hint about the location of the tiger that was $70\%$ accurate, via a growl behind the left door (GL) or a growl behind the right door (GR). The OL/OR actions opened the chosen door. After the participant opened a door, they saw the result and the system randomly reallocated the location of both tiger and reward ($50\%$ chance of being behind any particular door). Participants understood the underlying probabilities via task instructions and pre-task training and saw the potential reward associated with each action during each round, via the payout matrix. (see the task subsection in the methods section for a more detailed description of the task. Also see Fig. 1 for the setup and Figs. 2 and 3 for a depiction of the experimental presentation). The task differed in complexity depending on whether it was the single-agent version (TT) or the multiagent version (i.e., ITT). In the ITT participants also got social hints about the other participants’ actions with $80\%$ probability.

In the results section all the comparisons of the mean are done with the use of the Welch two-sample t-test. Briefly, the Welch test is a variant of the t-test that calculates effective degrees of freedom to correct for heteroscedasticity. For a detailed report on the statistics used in the paper please refer to the statistical analysis at the end of the methods section.

### Single-agent version

**Figure 4:**
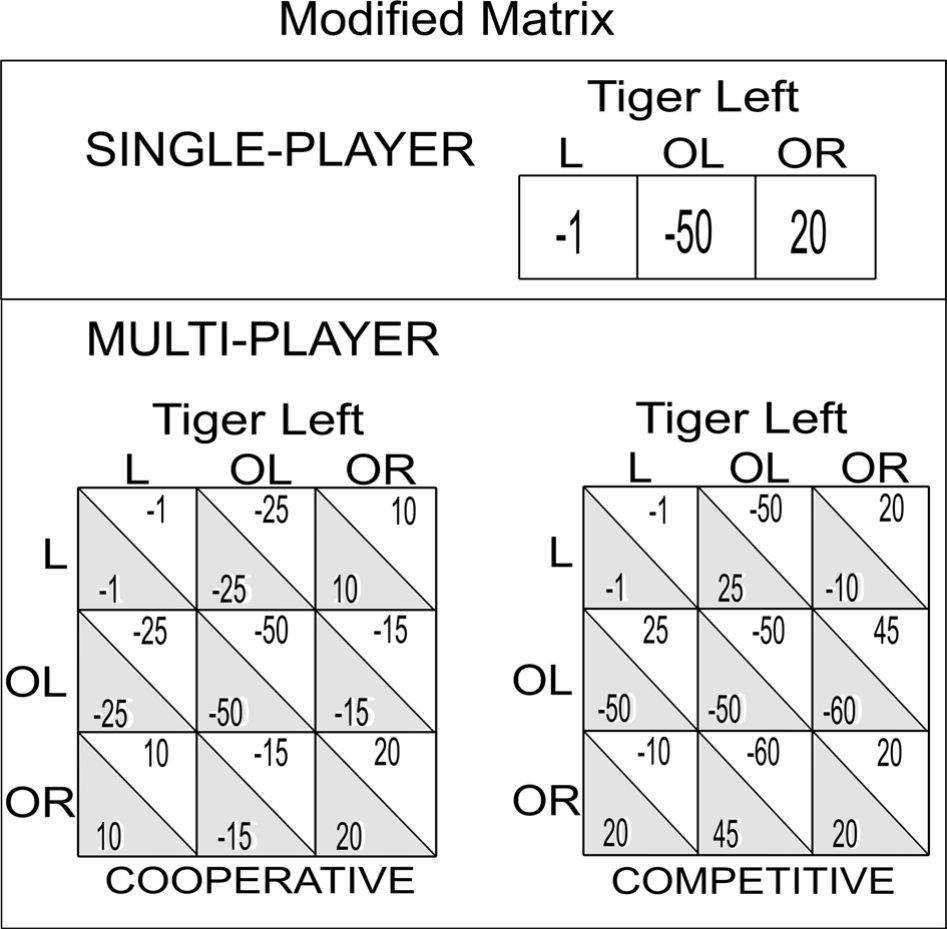
Modified payout structure of the Tiger Tasks. The payouts are potential points that can be gained for a chosen action (listen (L) and open-left/right (OL/OR)), when the tiger is behind the left door. The points scheme remains the same for the tiger behind the right door if we switch the OL and OR columns and rows. In the single-participant setting, a L action costs -1 points. Getting the gold-pot rewards +20 points while encountering the tiger takes away -50 points. In the multiagent setting, the column actions represent one’s own actions, while the row is the other participant’s actions. The point system is similar from the single-participant setting but more context dependent combinations are added as the other participant’s actions affect the points a participant can make.

**Figure 5:**
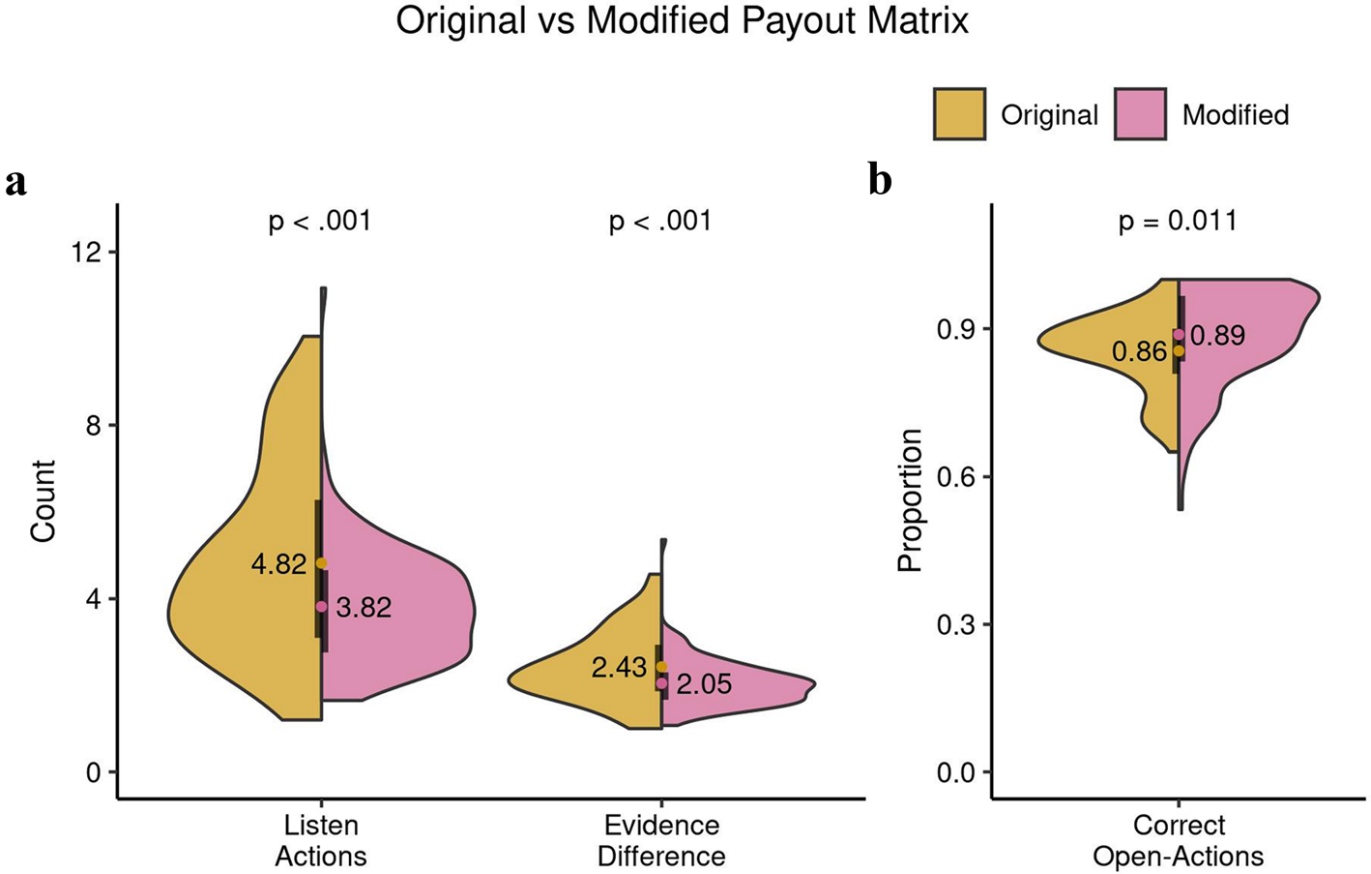
Behavioral indices: Comparison of the single-agent versions of the TT. This Figure compares the participants’ performance difference in the original version with the modified version of the payout matrix. It shows the mean values with their distributions in the form of the split violin plot. The black bars cover the interquartile range. In (**a**), we see the number of listen actions and the evidence-differences are significantly lower in the modified version. In (**b**), the number of correct open actions are higher in the modified version.

Three indices measured performance on the single-agent version of the tiger task (TT): (1) the number of listen actions, (2) the evidence-difference, and (3) the percentage of correct open actions (see methods section for details). Because every participant completed the TT and this version only contains physical observations (tiger growls) without the complexities of social interactions and mentalizing, it provides the best context for studying the effects of the original and modified version of the payout matrix. For the definition and difference between the two task versions please refer along with Fig. 4, to the methods section. Figure 5 shows the results.

Participants differed ($t(90.96) = 3.45, p = 0.86\times 10^{-3},$ $95\%$ CI [0.43, 1.58], Cohen $d=0.56$) in the number of listen actions between the two different versions of the TT. Participants in the modified version ($mean\pm s.d.$ : $3.82\pm 1.31$) required significantly fewer listen actions than in the original version ($4.82\pm 2.16$) before committing an open action. Participants showed a smaller evidence-difference in the modified ($2.05\pm 0.55$) compared to the original TT ($2.43\pm 0.80$, $t(98.19) = 3.48, p = 0.76\times 10^{-3},$ $95\%$ CI [0.16, 0.60], Cohen $d=0.56$). This is likely reflective of the larger risk associated with the original TT due to a larger loss following an incorrect open action (see Fig. 5a). Participants performed better in the modified version, judging by the number of correct open actions ($t(158.89) = -2.57, p = 0.011,$ $95\%$ CI $[-0.59, 0.01],$Cohen $d=0.38$; See Fig. 5b).

**Figure 6:**
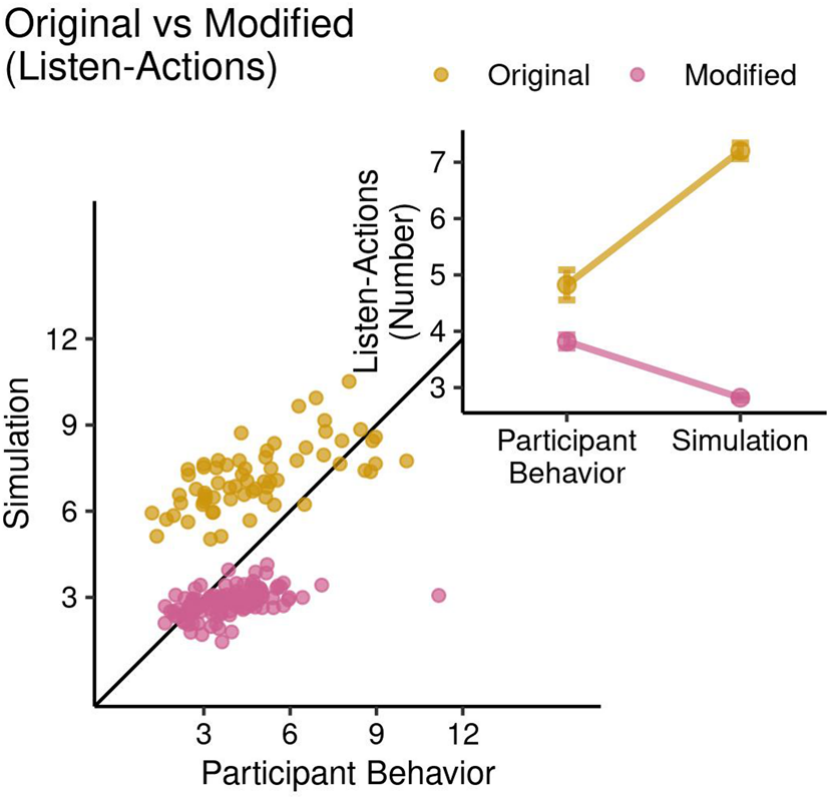
Comparison of actual and optimal number of listen action in the single-agent TT. Each dot represents a participant characterized by their actual (x-axis) and optimal (y-axis) number of listen actions averaged across all tiger trials. Whereas the optimal POMDP Level 1 model overestimates the number of listen action for the original payout matrix, it underestimates L actions for the modified payout matrix. In figure inset, we see main effects of the type of matrix, the participant behavior/simulation, and the interaction effect between them.

Participants’ choices deviated strongly from computationally optimal actions and there was a stark difference between the original and the modified payout matrix in the direction of the deviation (see Fig. 6.). The optimal POMDP model is defined in the methods section. For the original payout matrix the optimal POMDP solution consistently overestimated the participants’ number of listen actions (mean nListen: POMDP $7.20\pm 1.14$, participants $4.82\pm 2.16$, $t(98.29) = -7.91, p = 3.97\times 10^{-12},$ $95\%$ CI $[-2.98, -1.78],$Cohen $d=0.97$). However, for the modified payout matrix the POMDP underestimated the participants’ actual number of listen actions (mean nListen: POMDP $2.82\pm 0.46$, participants $3.82\pm 1.31$, $t(152.71) = 8.01, p = 2.67\times 10^{-13},$ $95\%$ CI [0.75, 1.24], Cohen $d=0.72$). Using a 2-way mixed-effects ANOVA with the within-subject factor as participant behavior and optimal POMDP, and the between-subject factor as the original and modified matrix, we observed the main effect of the type of matrix ($F(1,374) = 308.41, p < 2.00\times 10^{-16}$), the participant behavior and simulation ($F(1,374) = 24.33, p < 1.22\times 10^{-6}$), and also an interaction effect between them ($F(1,374) = 121.76, p < 2.00\times 10^{-16}$) (see Fig. 6. inset).

### Multiagent version

In the main text of this paper we focus on the results with the modified payout matrix of the multiagent ITT. All subsequent findings in this section therefore, refer to the modified ITT.

#### Task performance in participants and computational models

One of our main goals of this paper is to compare participants’ choice behavior in the ITT to that of I-POMDP models, which embody the optimal solution for social decision-making under partial observability of the physical and social information. Two important variables that define the complexity and computational tractability of I-POMDPs are the level of recursive ToM and the planning horizon used in the value iteration algorithm. A formal description of the I-POMDP framework including details on the level and planning horizon can be found in the Methods section.

We defined two I-POMDP models: a Level 1, Horizon 1 model (L1H1) and a Level 1, Horizon 2 model (L1H2), which differ in the planning horizon of 1 step. Both models assume Level 1, which means that the counterpart is modeled as an unintentional Level 0 player. We simulated each participant with both models by presenting the physical and social observations that the participant made (Growls and Creaks) to the model and then compared the overall performance and various choice indices between participants and both models.

**Figure 7:**
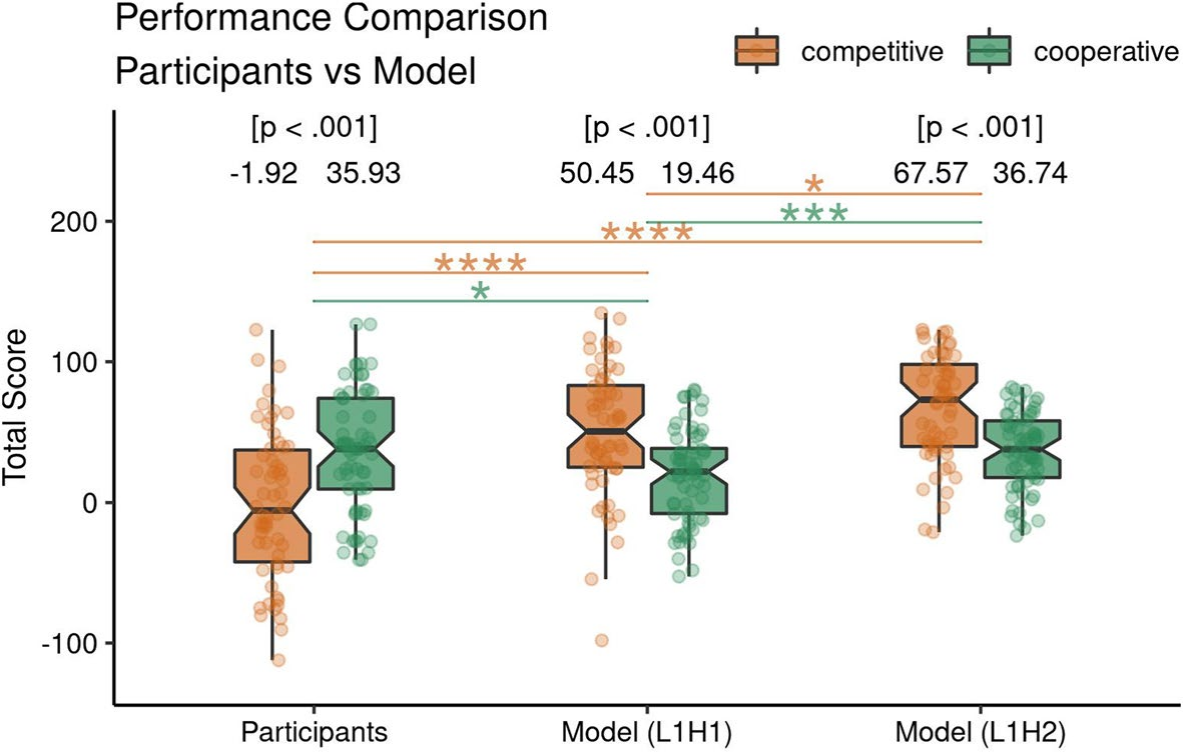
Comparison of performance of the participants with the models during the multiagent version of the ITT in competitive and cooperative contexts. The figure shows the points obtained (respective means above the box-plot) by the participants and the L1H1 and L1H2 optimal I-POMDP models. The points are individual participants in the respective contexts, while the p values demonstrating significance is above the means.

We observed a difference in the Total Score that participants and models received at the end of the experiment (see Fig. 7). In the competitive context, both models outperformed the participants suggesting that the way participants approached this version of the ITT represents a suboptimal solution that leads to smaller overall payoffs (participant vs L1H1 model $t(111.57) = -5.65, p = 1.248\times 10^{-7},$ $95\%$ CI $[-70.73, -34.00],$Cohen $d=0.74$; participant vs L1H2 model $t(102.46) = -8.09, p = 1.289\times 10^{-12},$ $95\%$ CI $[-86.53, -52.45],$Cohen $d=1.06$). However, in the cooperative context participants outperformed the L1H1 model ($t(122.26) = 2.46, p = 0.015,$ $95\%$ CI [3.21, 29.72], Cohen $d=-0.30$) and were equal to the L1H2 model ($t(109.63) = -0.13, p = 0.897,$ $95\%$ CI $[-13.23, 11.60],$Cohen $d=0.02$). At a first glance these initial findings suggest that participants in the competitive context maybe parsing the information provided in the payoff matrices, which induces the competitive context, differently than the two I-POMDP models. However, in the cooperative context the participants may be using a similar planning horizon to achieve comparable performance with the L1H2 model.

#### Interactive effects on choice indices

**Figure 8:**
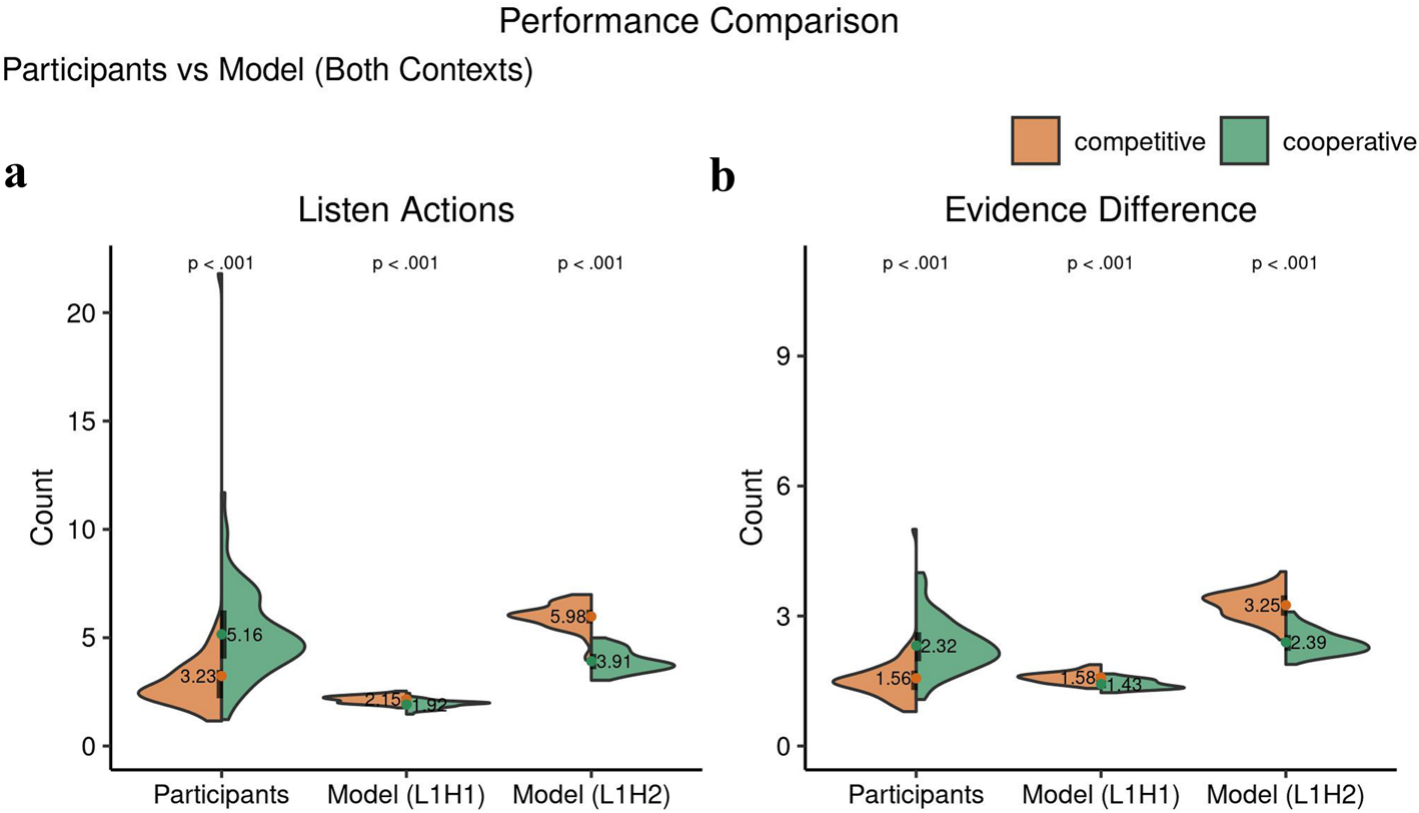
Interactive choice behavioral indices’ (listen actions and evidence-difference) comparison of the multiagent version of the ITT between participants and IPOMDP models, competitive and cooperative contexts. The figure shows the mean values with their distributions in the form of split violin plots. The black bars cover the interquartile range. The number of listen actions are significantly higher in the cooperative context for participants and the converse is true for both the IPOMDP models as seen in (**a**). Similar relations are observed in (**b**) for participants and both models for evidence-difference.

To compare the participants’ performance with the models and within the interactive context we further examined the effect of choice indices (defined in detail in the methods section). ITT condition strongly affected the number of listen actions ($t(102.49) = -4.54, p = 1.6\times 10^{-5},$ $95\%$ CI $[-2.76, -1.08],$Cohen $d=0.82$), which was higher in cooperation ($mean \pm s.d.$: $5.16 \pm 1.93$) than in competition ($3.23 \pm 2.67$) for participants, but the other way around for the two I-POMDP models (Model L1H1: competition -> $2.15 \pm 0.17$, cooperation -> $1.92 \pm 0.18$) ($t(121.36) = 7.42, p = 1.73\times 10^{-11},$ $95\%$ CI [0.17, 0.30], Cohen $d=1.33$) (Model L1H2: competition -> $5.98 \pm 0.60$, cooperation ->$3.91 \pm 0.15$) ($t(110.13) = 20.74, p = 7.69\times 10^{-40},$ $95\%$ CI [1.87, 2.26], Cohen $d=3.76$) (see Fig. 8a). This suggests that cooperative participants engaged in prolonged evidence gathering in forming their estimates of the tiger location. The number of listen actions in the cooperative ITT was higher than in the single agent TT ($t(97.59) = -5.04, p = 2.155\times 10^{-6},$ $95\%$ CI $[-1.86, -0.81],$Cohen $d=0.57$), with no difference between TT and competitive ITT ($t(70.13) = 1.58, p = 0.12,$ $95\%$ CI $[-0.15, 1.32],$Cohen $d=0.20$). Under cooperation, participants knew that maximal reward was achieved by coordinated open actions, and so they would not have had incentive to race one another to the door. Conversely, under competition, participants were expected to view the task as a race so as to beat the other participant to the correct door and receive maximal reward. That is, participants would have likely underestimated their own probability of opening the wrong door by overestimating the weight of loss if their competitor would have opened the correct door. The models on the other hand gathered more evidence before opening the door rather than take it as a race to the door.

This was paralleled by a significant effect on evidence gathering difference between interactive contexts. Evidence difference is the number of observations from the tiger side minus the observations from the other side (see methods section for details). In the competitive context, the participants preferred to open the door with less evidence gathering ($1.56 \pm 0.57$) than in the cooperative context ($2.32 \pm 0.60$) ($t(121.14) = -7.12, p = 8.2\times 10^{-11},$ $95\%$ CI $[-0.96, -0.54],$Cohen $d=1.28$), pointing towards increased coordination during cooperation and increased risk-taking during competition. While the two I-POMDP models show the converse effect inline with the number of listen actions (Model L1H1: competition: $1.58 \pm 0.13$, cooperation: $1.43 \pm 0.11$) ($t(113.06) = 7.29, p = 4.46\times 10^{-11},$ $95\%$ CI [0.11, 0.20], Cohen $d=1.32$) (Model L1H2: competition -> $3.25 \pm 0.32$, cooperation -> $2.39 \pm 0.27$) ($t(111.25) = 16.11, p = 7.62\times 10^{-31},$ $95\%$ CI [0.75, 0.296], Cohen $d=2.92$) (see Fig. 8b). Comparing the evidence gathering difference of the participants in ITT to the TT, we saw a significantly lower difference in competition ($t(58.83) = -3.80, p = 3.5\times 10^{-4},$ $95\%$ CI $[-2.05, -0.63],$Cohen $d=0.61$) and a significantly greater difference in cooperation ($t(69.09) = -12.49, p < 2.2\times 10^{-16},$ $95\%$ CI $[-3.50, -2.53],$Cohen $d=0.33$).

**Figure 9:**
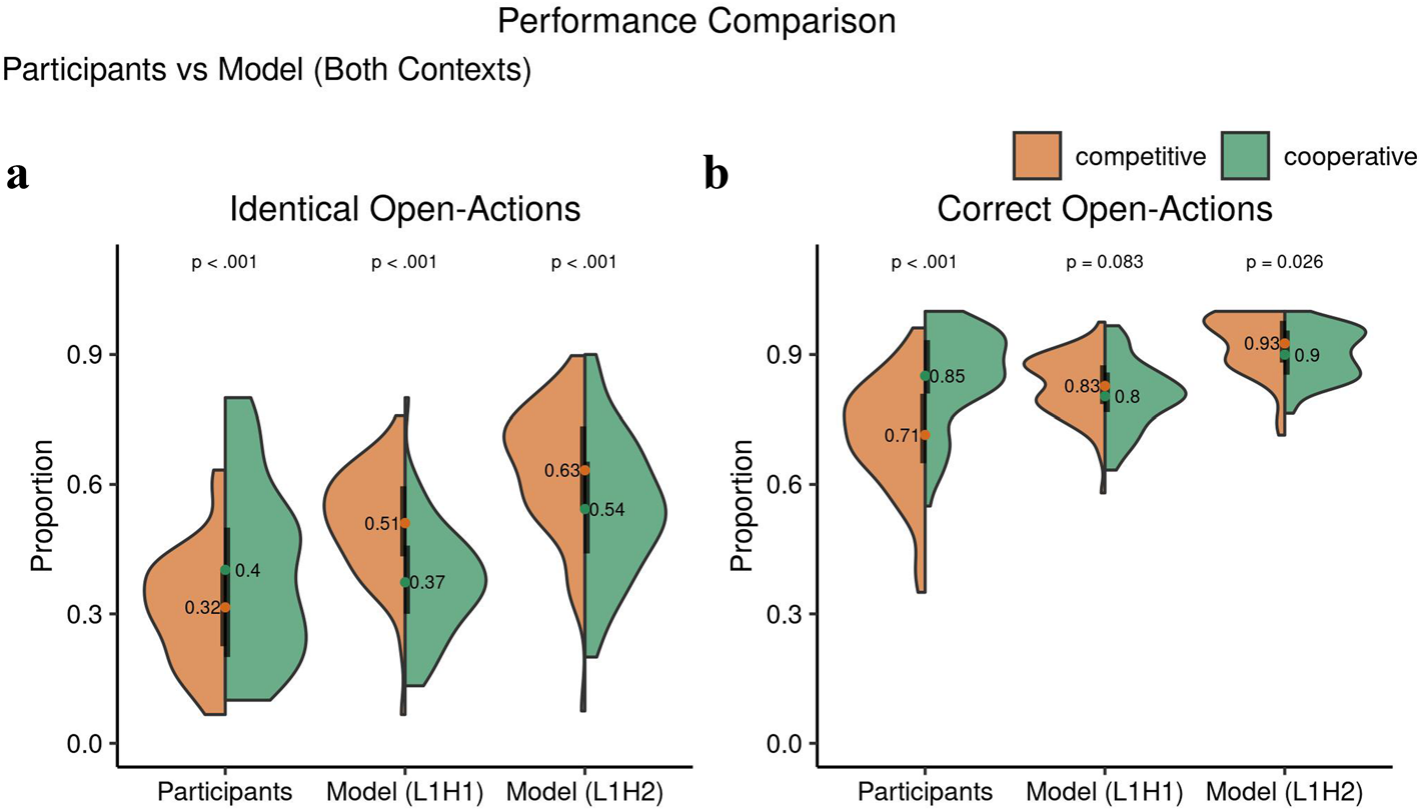
Interactive choice behavioral indices’ (identical and correct open actions) comparison of the multiagent version of the ITT between participants and IPOMDP models, competitive and cooperative contexts. The figure shows the mean values with their distributions in the form of split violin plots. The black bars cover the interquartile range. In (**a**) we observe that identical open actions are significantly higher in cooperative context for participants, however in the IPOMDP models it is higher in the competitive context. In (**b**) comparing the correct open actions we observe similar behavior for the participants and the IPOMDP model L1H2, however for the Model L1H1 there is no significant difference between the contexts.

We further observed a higher percentage of identical open actions in the cooperative ($0.40 \pm 0.20$) compared to the competitive context ($0.32 \pm 0.13$, $t(113.36) = -2.86, p = 4.9\times 10^{-3},$ $95\%$ CI $[-0.15, -0.03],$Cohen $d=0.51$) suggesting that participants in the cooperative context, which incentivized action coordination, increased successful coordination. The I-POMDP models showed increased coordination in the competitive context as well that led to better performance (Model L1H1: $0.51 \pm 0.12$; Model L1H2: $0.63 \pm 0.15$) (please refer to the section explaining the task performance). Additionally, the model behavior was contextually reversed with less identical open actions in the cooperative versions (Model L1H1: $0.37 \pm 0.13$; Model L1H2: $0.54 \pm 0.16$) (Context difference for Model L1H1: $t(120.44) = 6.07, p = 1.50\times 10^{-8},$ $95\%$ CI [0.09, 0.18], Cohen $d=1.09$; Model L1H2: $t(121.16) = 3.24, p = 1.56\times 10^{-3},$ $95\%$ CI [0.03, 0.14], Cohen $d=0.58$) (see Fig. 9a).

Finally, we also found a significant difference in the percentage of correct open actions, which was higher during cooperation ($0.85 \pm 0.11$) than competition ($0.71 \pm 0.13$, $t(111.55) = -6.38, p = 4.2\times 10^{-9},$ $95\%$ CI $[-0.18, -0.09],$Cohen $d=1.15$). This suggests that participants in the cooperative context might have been able to utilize their longer evidence gathering (number of listen actions) effectively for more successful open actions. The I-POMDP models have significantly better correct open actions in the competitive context (Model L1H1: $t(89.98) = -5.83, p = 8.52\times 10^{-8},$ $95\%$ CI $[-0.15, -0.07],$Cohen $d=-1.08$; Model L1H2: $t(87.76) = -11.05, p = 2.69\times 10^{-18},$ $95\%$ CI $[-0.25, -0.17],$Cohen $d=-2.05$) which maybe assisting in better performance than the participants, however in the cooperative context the Model L1H1 has less correct open actions than the participants ($t(119.33) = 2.89, p = 5.00\times 10^{-3},$ $95\%$ CI [0.01, 0.08], Cohen $d=-0.50$), while Model L1H2 has more ($t(99.43) = -3.28, p = 1.00\times 10^{-3},$ $95\%$ CI $[-0.08, -0.02],$Cohen $d=-0.57$), which significantly impacts the overall points obtained (refer to the section on task performance) (see Fig. 9b).

#### Variables affecting the differences in task performance

What task variables account for the performance difference between participants and models in the competitive context? We addressed this question with a linear mixed-effects regression, which included as predictors the number of Listen actions (nListen), the number of correct Open actions (CorrOpen) and the number of identical Open actions (IdentOpen) of both agents in the dyad, and the ’group’ variable that codes for participants, L1H1, and L1H2 models. As random effects we included a random intercept for each participant (or modeled subject in L1H1 and L1H2). Model comparison of various version of this model (see Supplement) reveals that a version with all interactions provided the best fit to the data (see Table s2 and s3 (supplement) for model comparison values).

Group membership (participants, L1H1, L1H2) had a significant effect in the competitive context reflecting the differences in Total Score between participant and both models.

**Figure 10:**
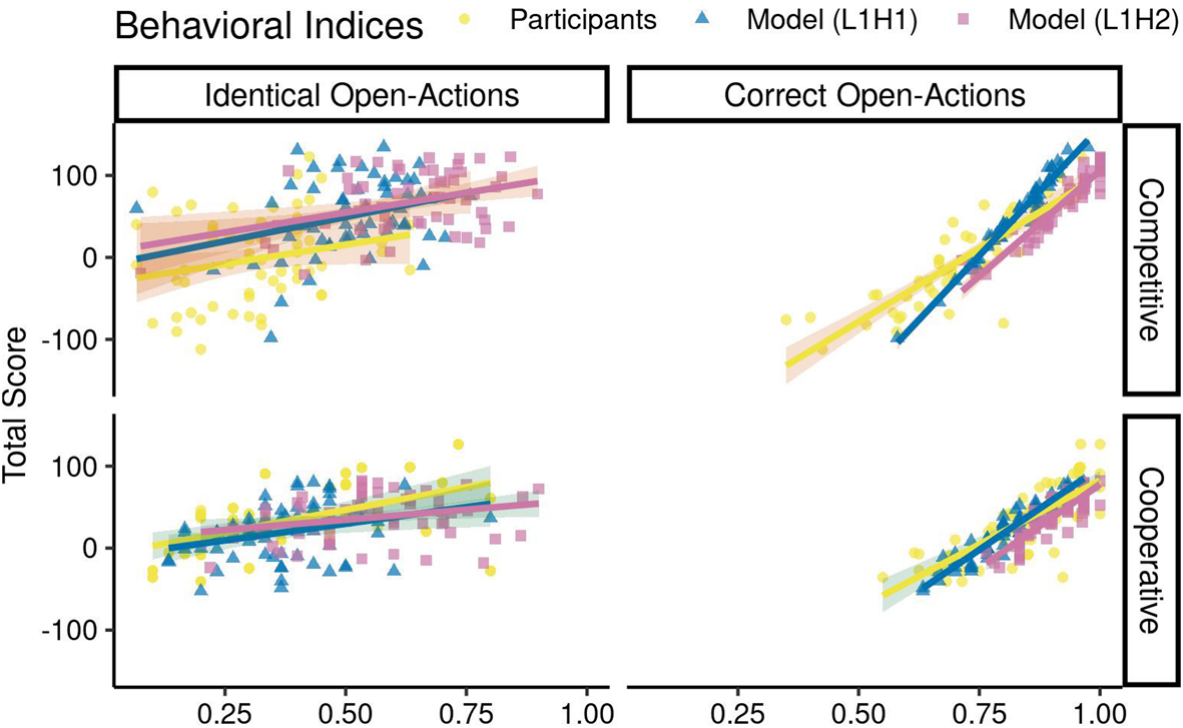
Linear mixed-effects regression model comparison between the participants and the two IPOMDP models. Correct open-actions and identical open-actions have significant effect on the total score in both context.

In both context we found a highly significant main effects for CorrOpen (Competitive: beta = 463.71, $95\%$ CI [225.76, 701.66], $t(154.99) = 3.82, p < .001$; Cooperative beta = 205.24, $95\%$ CI [52.69, 357.79], $t(162.28) = 2.64, p = 0.008$) (see Table s3 (supplement) for a list of significant effects in this linear model in both interactive contexts). Furthermore, group differences in CorrOpen had a significant effect on explaining the variation in Total Score in the competitive context (L1H1: beta = 122.86, $95\%$ CI [51.73, 194.00], $t(139.91) = 3.39, p < .001$; L1H2: beta = $-69.74$, $95\%$ CI $[-179.94, 40.46]$, $t(152.10) = -1.24, p = 0.215$), but not in the cooperative context (L1H1: beta = 35.72, $95\%$ CI $[-22.97, 94.42]$, $t(140.13) = 1.19, p = 0.233$; L1H2: beta = $-9.99$, $95\%$ CI $[-64.32, 44.34]$, $t(127.81) = -0.36, p = 0.719$). Both models achieved a higher number of CorrOpen than the participants during competition, but not during cooperation (see Fig. 10).

Moreover, IdentOpen exhibited a marginally significant effect during cooperation (beta $= -347.54$, $95\%$ CI $[-687.25, -7.83]$, $t(163) = -2.01, p = 0.045$) probably reflecting the fact that the maximal payoff in this context can be achieved if both players open the correct door at the same time. The interaction effect between IdentOpen and group was also significant both contexts (Competitive (L1H1): beta = $-42.68$, $95\%$ CI $[-85.52, 0.15]$, $t(131.47) = -1.95, p = 0.051$; Competitive (L1H2): beta $= 77.04$, $95\%$ CI [22.51, 131.58], $t(132) = 2.77, p = 0.006$; Cooperative (L1H1): beta = $-5.11$, $95\%$ CI $[-30.31, 20.08]$, $t(118.23) = -0.40, p = 0.691$; Cooperative (L1H2): beta = $-33.64$, $95\%$ CI $[-53.15, -14.14]$, $t(118.56) = -3.38, p < .001$), but the pattern of group differences in IdentOpen varied between contexts: during competition the models showed a higher effect of IdentOpen on the Total Score, whereas during cooperation the participant and the L1H2 model IdentOpen was more strongly related to Total Score than that of the L1H1 model.

Interestingly, the number of Listen actions was not important in explaining variation in the Total Score, neither as as main effect (Competitive: beta = 31.79, $95\%$ CI $[-11.41, 74.99]$, $t(135.41) = 1.44, p = 0.149$; Cooperative: beta $= 8.10$, $95\%$ CI $[-14.65, 30.86]$, $t(150) = 0.70, p = 0.485$) nor as an interaction with the group membership. Therefore, despite pronounced differences in nListen between participant and both models (Competitive (L1H1): $t(57.48) = 3.07, p = 3.00\times 10^{-3},$ $95\%$ CI [0.38, 1.78], Cohen $d=0.57$; Competitive (L1H2): $t(62.79) = -7.63, p = 1.58\times 10^{-10},$ $95\%$ CI $[-3.46, -2.03],$Cohen $d=-1.42$; Cooperative (L1H1): $t(66.17) = 13.56, p = 9.05\times 10^{-21},$ $95\%$ CI [2.76, 3.72], Cohen $d=2.36$; Cooperative (L1H2): $t(73.37) = 5.07, p = 2.93\times 10^{-6},$ $95\%$ CI [0.75, 1.73], Cohen $d=0.88$) these were not important for explaining the variation in performance of the competitive and cooperative ITT.

This difference was explained by CorrOpen and IdentOpen in the competitive context. The difference in between the participants and both models (Competitive (L1H1) CorrOpen: $t(89.98) = -5.83, p = 8.52\times 10^{-8},$ $95\%$ CI $[-0.15, -0.07],$Cohen $d=-1.08$; Competitive (L1H2) CorrOpen: $t(87.76) = -11.05, p = 2.69\times 10^{-18},$ $95\%$ CI $[-0.25, -0.17],$Cohen $d=-2.05$; Competitive (L1H1) IdentOpen: $t(113.55) = -8.18, p = 4.62\times 10^{-13},$ $95\%$ CI $[-0.24, -0.15],$Cohen $d=-1.52$; Competitive (L1H2) IdentOpen: $t(112.13) = -12.05, p = 6.05\times 10^{-22},$ $95\%$ CI $[-0.37, -0.27],$Cohen $d=-2.24$) culminated in better score for the models than the participants, and model L1H2 better than model L1H1 (CorrOpen: $t(113.80) = -7.50, p = 1.49\times 10^{-11},$ $95\%$ CI $[-0.12, -0.07],$Cohen $d=-1.39$; IdentOpen: $t(110.01) = -4.77, p = 5.77\times 10^{-6},$ $95\%$ CI $[-0.17, -0.07],$Cohen $d=-0.89$;) (see Figs. 7 and 9). However, to understand the contributors in the total score we ran two more linear mixed-effects regression models and observed that nListen predicts the CorrOpen (beta = 0.08, $95\%$ CI [0.05, 0.12], $t(178.59) = 4.55, p = 9.86\times 10^{-6}$) and IdentOpen (beta = 0.07, $95\%$ CI [0.01, 0.13], $t(159.64) = 2.39, p = 0.018$) significantly. This finding indicates that the combination of the CorrOpen and IdentOpen predicted the total score in the cooperative context and these were themselves predicted by the number of Listen actions.

In summary, these analyses demonstrate that participants perform sub-optimal during the competitive ITT, but that they are almost as good as the L1H2 model during cooperation. Group differences in Correct Open action and Identical Open action are relevant for explaining the differences in overall task performance.

#### Interactive effects on prediction indices

**Figure 11:**
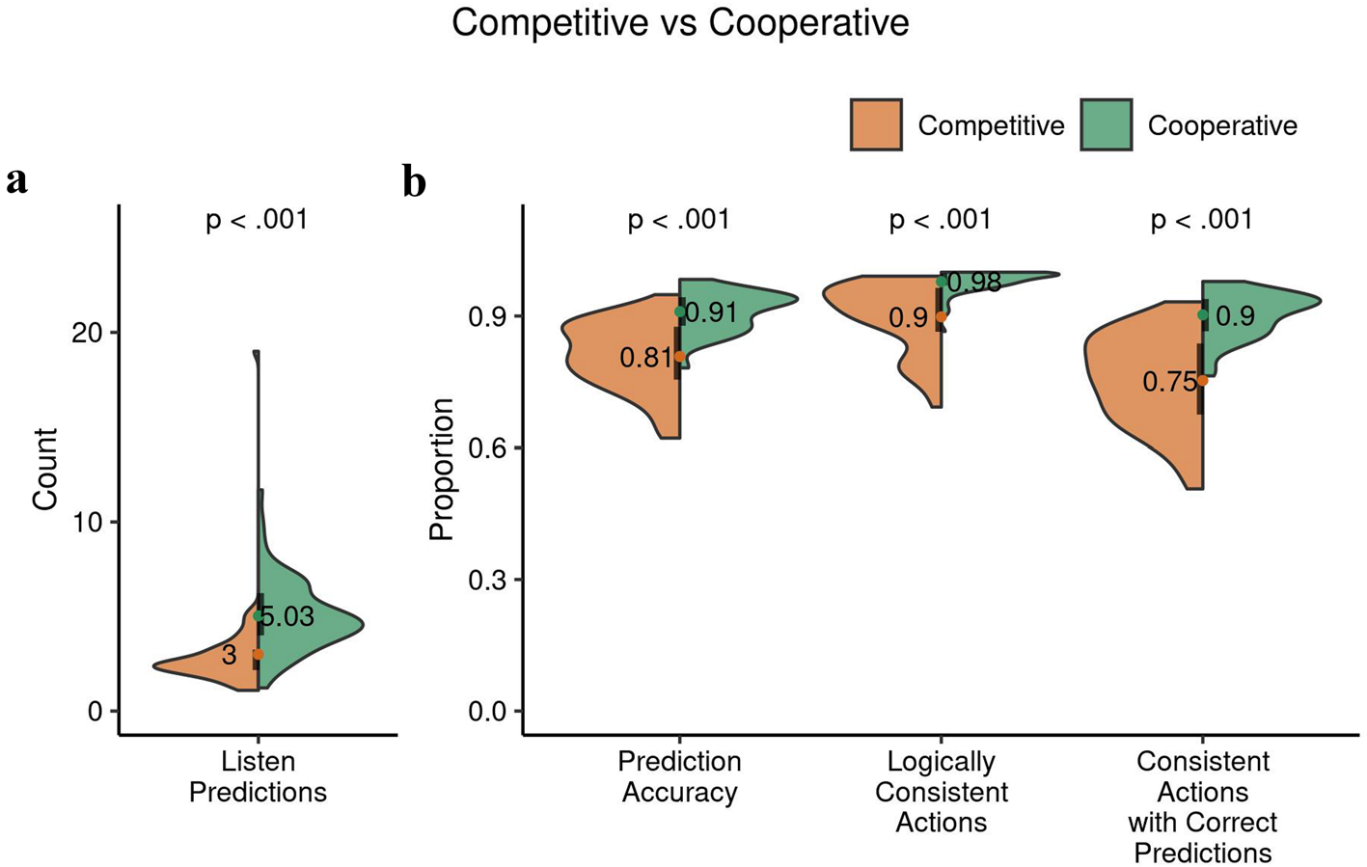
Behavioral indices of prediction: comparison of the competitive and cooperative ITT. Mean values with their distributions in the form of split violin plots. The black bars cover the interquartile range. In (**a**), the number of listen predictions are significantly higher in the cooperative context. In (**b**), the higher prediction accuracy in the cooperative ITT indicates that the participants were better able to anticipate others’ choice behavior. Logically consistent actions and consistent actions following correct predictions of others’ choices are important criteria in achieving favorable outcomes in the cooperative context, as seen with significantly higher percentages during cooperation.

In the next step we examined the effects of the interactive context on behavioral indices of predictions (see Fig. 11, and refer to methods for the indices description). These analyses also shed some light on the cognitive processes involved in modeling the beliefs and choices of the other participant. We first compared prediction accuracies in both interactive contexts. Participants exhibited more accurate predictions of the other participant in the cooperative context ($0.91 \pm 0.45$) compared to the competitive context ($0.81 \pm 0.08$, $t(89.47) = -8.82, p = 8.6\times 10^{-14},$ $95\%$ CI $[-0.13, -0.08]$, Cohen $d=1.61$). Participants likely responded to the demand characteristics of the cooperative ITT, which incentivizes precise coordination of actions and hence a demand for accurate predictions of the other participant’s actions (see Fig. 11a).

**Figure 12:**
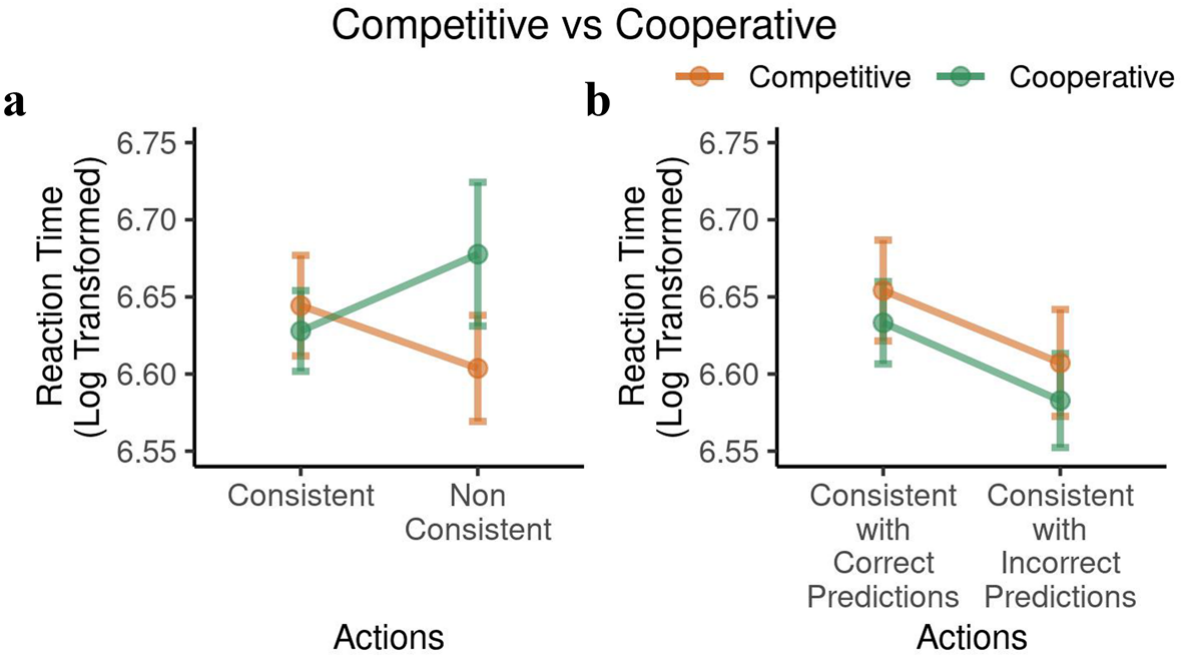
Comparison of reaction times (RTs) based on logical consistency of actions. Using 2-way mixed effects ANOVA we observed no interaction effect of the context and the consistency of actions in (**a**). Using 2-way mixed effects ANOVA we observed a main effect of accuracy, and no interaction effects in (**b**) between the correctly and incorrectly predicted consistent actions. All error bars show mean +/- the standard error.

Paralleling the higher number of listen actions in the cooperative ITT, we also observed a higher number of listen predictions during cooperation ($5.03 \pm 1.87$) than during competition ($3.00 \pm 2.34, t(109.04) = 5.29, p = 6.42\times 10^{-7},$ $95\%$ CI $[-2.80, -1.27],$Cohen $d=0.96$). As cooperating participants demonstrated longer evidence gathering before an open action, they may have projected the same tendency onto their co-participants, or they may have developed expectations from their partial observations of the other participant’s listen actions (see Fig. 11b).

Similarly, we also found a higher percentage of logically consistent actions during cooperation ($0.98\pm 0.03$) than competition ($0.90 \pm 0.08$, $t(67.79) = -7.50, p = 1.8\times 10^{-10},$ $95\%$ CI $[-0.10, -0.06],$Cohen $d=1.38$). This is consistent with an expectation that participants would act more predictably during cooperation than competition. Moreover, we also observed that among the consistent actions, those that were correctly predicted for the other participant were higher during cooperation ($0.90 \pm 0.05$) than competition ($0.75 \pm 0.10$, $t(80.77) = -10.06, p = 6.8\times 10^{-16},$ $95\%$ CI $[-0.18, -0.12],$Cohen $d=1.85$) (see Fig. 11b).

Next we compared the reaction times (RTs) for consistent and non-consistent action in both interactive contexts. After log-transforming the RTs to approximate a Gaussian distribution, we used a 2-way mixed-effects ANOVA with the within-subject factor action consistency and the between-subject factor context. We did not observe any main effect of consistency ($F(1,115) = 0.08, p = 0.78$) or interaction effect of consistency with ITT condition ($F(1,115) = 3.24, p = 0.074$) (see Fig. 12a).

Subsequently, we sub-divided RTs for consistent actions into those associated with correct and incorrect predictions in both interactive contexts. A 2-way mixed-effects ANOVA showed a main effect of prediction accuracy (correctly predicted consistent actions: $6.64 \pm 0.23$, incorrectly predicted consistent actions: $6.59 \pm 0.25$, $F(1,122) = 15.42, p = 0.143\times 10^{-3}$), but no main effect of context ($F(1,122) = 0.29, p = 0.592$) and no interaction ($F(1,122) = 0.02, p = 0.888$) (see Fig. 12b). This is consistent with the notion that accurate mentalizing about the other participant, which likely leads to more accurate predictions, results from deeper engagement of cognitive resources, leading to longer RTs regardless of the interactive context.

#### Relating choice and prediction indices

**Figure 13:**
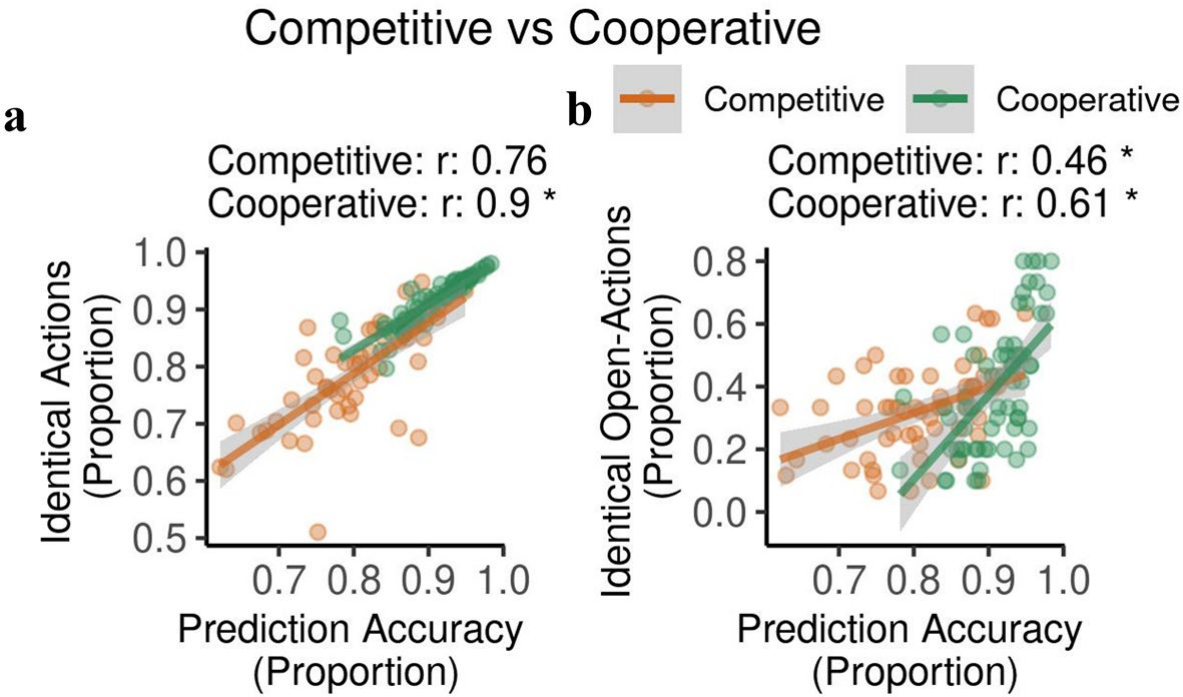
Correlations relating choice and prediction indices. We observed significant correlation between (**a**) prediction accuracy and identical actions with significance between the contexts. We also observed significant correlation between (**b**) prediction accuracy and identical open actions.

To answer the question of whether better prediction accuracy resulted in improved dyadic coordination and in improved overall performance, we related the differences in prediction performance in the two contexts to the choice performance of our participants (Fig. 13). Prediction accuracy correlated with identical action indices across all three action types (cooperative: $r=0.90,$ $95\%$ CI [0.84, 0.94]; competitive: $r=0.76,$ $95\%$ CI [0.62, 0.85]) suggesting that better prediction performance improved the coordination of actions. We observed significant correlation coefficients in both contexts, nevertheless, the correlation coefficient is significantly higher during cooperation than competition ($z_\text {observed}=-2.55,p < 0.05$) (Fig. 13a) indicating significant advantage of predicting accurately during cooperation. Indices for identical open actions also correlated with prediction accuracy (cooperative: $r=0.61,$ $95\%$ CI [0.43, 0.74]; competitive: $r=0.46,$ $95\%$ CI [0.22, 0.64]), but there was no difference ($z_\text {observed}=-1.14$) due to ITT condition (Fig. 13b).

#### Learning-related improvement in task performance

**Figure 14:**
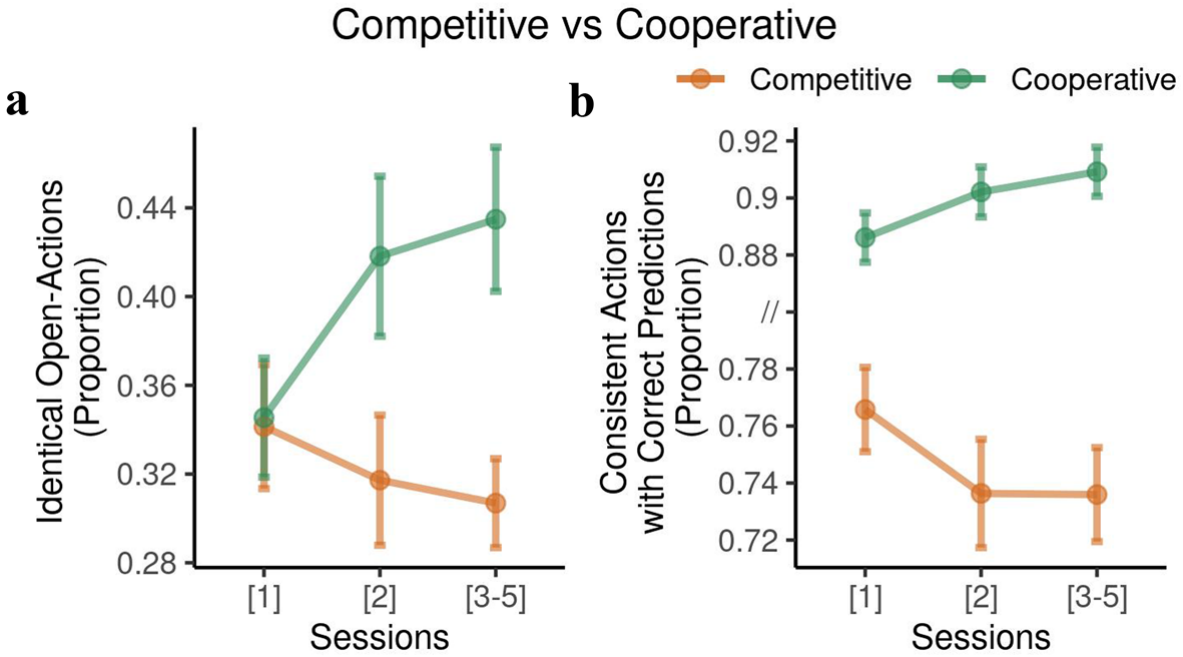
Learning effect in the interactive Tiger Task. Using 2-way mixed effects ANOVA, in (**a**) we observed a main effect of context and a significant interaction indicating that participants in the cooperative context learn to simultaneously open the door through subsequent sessions, compared to the competitive context. For the correctly predicted consistent actions in (**b**) we see a similar effect of context and session. Bars show standard errors.

We expected that participants would learn about the other participants’ preferences and beliefs through repeated interactions in the ITT. Learning should change behavioral indices across all three sessions. Figure 14. shows that most learning occurred between sessions 1 and 2.

Paralleling our findings on identical actions in Fig. 9., we saw a main effect of cooperation vs. competition on identical open actions ($F(1,122) = 6.04, p=0.015$). Cooperation yielded higher numbers of identical open actions that increased over sessions (interaction effect context x session $F(2,244) = 3.69, p = 0.026$). The number of consistent actions with correct predictions increased slightly during the cooperative ITT and sharply decreased during the competitive ITT (interaction effect context x session, $F(2,244) = 4.16, p = 0.017$; main effect for competitive ITT, $F(1,122) = 115.2, p<2\times 10^{-16}$). This is consistent with the expected demand characteristics of the two versions of the ITT.

## Discussion

The Tiger Problem is an iconic challenge as AI seeks to develop sophisticated models for planning under uncertainty and especially seeks to achieve adaptive interactions with human agents^22,27^. Tiger Problem models capture the belief updating process, in which the agent has only partial access to information about the current state that is relevant to probabilistic rewards, manipulating the factors of both interactivity and uncertainty^25^.

In the TT, we found that reduced risk/reward ratio in the modified payout matrix increased how well the optimal model matched the number of listen actions from human agents (Fig. 6). This is consistent with our hypothesis that participants underestimated their probability of loss while overestimating the weight of the small losses incurred by gathering evidence^30,31^.

As expected we found higher performance during cooperation for participants on most of our indices for the ITT while the models showed the converse effect in the choice indices (Figs. 8, 9 and 11). Models show that in the competitive context it is beneficial to have higher number of correct open actions to accumulate a higher score. While, in the cooperative context, the number of listen actions play a significant part in predicting the identical open actions and correct open action, and a combination of these impact the total score (Figs. 7 and 10).

We see participants gather more evidence during cooperation compared to competition on the choice indices, while both models do not. Thus, unlike human participants under competition, optimal models do not race to open a door. Rather, optimal models during competition continue to gather evidence that translates into a higher score.

Cooperation elicited more evidence gathering and logically consistent actions, along with better predictions and outcomes for the participants. Better consistency yields better predictability, and so the measures could reveal an interesting step in a recursive hierarchy of ToM processes, where “I think that you think...” becomes “I think that you think that I think...” and so on. Increasing one’s own predictability allows greater likelihood of coordination and cooperation. This may indicate that cooperative participants represented the other participant’s perspective. Strict coordination of open actions requires accurately assessing when one’s own belief matches the co-participants’ belief. This would be achieved by prolonged evidence gathering, leading to an increased evidence-difference observed in the cooperative ITT.

A further interesting aspect of the data is that during cooperation, participants exhibited shorter reaction times for consistent compared to non-consistent actions. This would be expected if consistent actions require less cognitive effort, since they are aligned with their strategic goals in the cooperative ITT (namely to be predictable in their action selection). Non-consistent actions violate these goals, which may be the reason why it takes more effort and time to make them. However, the RT analyses also revealed that among the consistent actions, people took longer when correctly predicting actions of others. This suggests that successful reasoning about other participants’ actions requires more cognitive resources and thus more time. There has been previous work exploring this in more detail^32^.

Participants learned during the ITT sessions. Namely, identical actions and correctly predicted consistent actions increased during cooperation but decreased during competition. These learning-related changes suggest that participants in both interactive contexts learned to adopt the incentives implied in the respective payout matrices. During cooperation participants learned to coordinate their actions and improve their own predictability as they learned to adapt to their co-participants, whereas during competition, participants learned to become less predictable.

In conclusion, our study is the first to provide empirical data from human participants as they engage in novel implementations of the single agent and multiagent, interactive Tiger Problem. The Tiger Problem has been essential for the generalization of Markov Decision Processes (MDPs) to more realistic settings of partial observability, in which information about the state of the world is only available through probabilistic observations^22^. Adding a social dimension to this Partially observable Markov Decision Process (POMDP) resulted in the framework of interactive POMDPs (I-POMDPs) for multiple, jointly interacting agents, whose actions are only available to each participant through probabilistic observations^26^. Both POMDPs and I-POMDPs resemble real world decision-making in a much more realistic way than conventional MDPs in that we often have to operate in a world of only partially available information. Thus, we believe that the empirical data generated from our implementations of the Tiger Problem will be of great interest to researchers in the modeling community who can use them as a reference for validating further development of these computational frameworks. In particular, our model-free findings can serve as targets for posterior predictive checks in future work with more detailed versions of POMDPs and I-POMDPs that can build “a model within a model” of the other participant. Similarly, our findings will be also relevant for researchers in decision (neuro)science who are interested in interactive decision-making under physical and social uncertainty and their neural underpinnings in newly designed, interactive tasks^33,34^. We observed that human participants departed from computationally optimal choices during competition but not during cooperation. This divergence has implications for understanding the complexity of cognitive and neural processes underpinning Theory of Mind in different social contexts.

Future attempts to move from computationally optimal models^8^ to applications with human persons and groups should proceed with caution and with empirical behavioral data. Otherwise, deployment of these technologies in real situations with real human beings will be less successful than is possible or will fail completely.

## Methods

### Participants

This study was approved by the Ethics Committee of the German Psychological Association (ref no. JG012015-052016) and carried out according to the Declaration of Helsinki. All participants gave written informed consent and were financially reimbursed for their participation. A total of 182 participants engaged several variants of the Tiger Task (TT and ITT), all of whom were naive to the tasks. The variants differed in their payout structure (original/modified, see below for details), their complexity (single/multiagent), and their interactive context (cooperative/competitive). From the total, 58 participants completed the original payout structure variant of the TT^22^, after which they completed either the cooperative (30 participants) or the competitive context (28 participants) with random assignment. All participants completed the single-agent version of the TT before the multiagent ITT version. The remaining 124 participants (82 women) took part in the modified payout structure variant of the TT and ITT. After participating in the TT, half the participants (randomly assigned) completed the cooperative ITT and the other half completed the competitive ITT. Women were aged $mean\pm s.d.$ : $25.30\pm 3.73$ years and men $24.88\pm 3.67$ years for the modified variant, while $24.75\pm 3.34$ and $25.32\pm 3.90$ respectively for the original variant.

### Experimental schedule

An ITT dyad consisted of 2 participants who were comfortably seated next to each other at two computer screens (eye-to-screen distance of 65 cm). A partition separated the participants so that each could only see their own screen. The experimenter was a muted observer for the duration of the task, separated from the two participants by an additional partition (Fig. 1). The room temperature averaged $mean\pm s.d.$ : $22.59\pm 1.31$ degrees Celsius. The dyad interacted using either the original payout structure or the modified payout structure of the ITT after participating in the respective TT. Each dyad completed either the cooperative or the competitive version of the ITT, but not both. The experimental setup was implemented in the Psychtoolbox Version 3.0.14^35^ running under MATLAB version 9.1.0 (The MathWorks, Natick, MA). Concurrent dyadic, synchronized high density EEG was recorded from a subset of participants, for use in subsequent analyses. Those will not be presented in this paper. Good performance also earned participants a relative financial bonus. The experimental code for running the experiment is available from the project’s github page https://github.com/SteixnerKumar/tiger_task_experiment.

### Task

A “session” of the TT or ITT for each participant was a total of 10 tiger-trials (i.e., 10 door openings). The participant sought to maximize the total rewards obtained during the task. In the ITT, L actions provided probabilistic information about both the tiger’s location and the other agent’s action. The sound of a creaking door on the left or right (CL or CR) gave partial evidence that the other agent opened the corresponding door and silence (S) gave partial evidence that no door was opened. These cues were $80\%$ accurate regarding the true action of the other agent. Additionally, the tasks used color coding to help differentiate actions of self (yellow) and other (blue).

#### Single-agent version

In the single-agent version, the participant completed the task alone. A “tiger-trial” consisted of a sequence of listen actions that ended with the participant opening one of the doors. During a session, the location of the tiger/reward changed only if a door was opened.

#### Multiagent version

The task began with a choice-screen (5*sec*.) asking the participant to choose an action (Fig. 2). The number of L actions was unlimited, but if a choice was not made during the allotted time, a forced listen action moved the task forward. Following an intra-event-interval (IEI)($+ \sim 1600ms.$), participants saw either the observation-screen (5*sec*.) after a listen action (L) or the outcome screen (5*sec*.) after an open-left (OL)/open-right (OR) action, which revealed the gain from the particular tiger-trial and the total gain from the session to that point. Opening the correct door (pot of gold) incurred a small win, whereas opening the incorrect door (tiger) incurred a large loss. The participant gathered sufficient evidence about the tiger’s location through a series of L actions (incurring a small loss each time) before opening a door (OL/OR action).

A given participant completed either a cooperative or a competitive ITT, but not both. To perform optimally in the task, participants had to use partial observations to update beliefs about the tiger’s location (i.e., growls (GL/GR)) and the other participant’s actions (i.e., creaks (CL/CR), silence (S)), along with representations of that participant’s beliefs and expected values. This combination of partial information made the ITT complex because the rewards were determined by joint actions of both participants. This complexity required higher cognitive loads and promoted reasoning about the other participant’s choices to develop a strategy for when best to open a door. In the cooperative context, the maximum joint reward occurred when both participants opened the correct door on the same round, while in the competitive context, the maximum payoff occurred when the participant opened the correct door while the other participant opened the wrong door on the same round.

We modified the original design of the multiagent Tiger Problem^26^ (Fig. 3) such that each round began with the prediction-screen (indicated by a blue dot) that required participants to predict the other participant’s action on that round (L, OL or OR). Next, the choice-screen (indicated by a yellow dot) prompted the participant for their own action (L, OL or OR), just as in the single-agent version. The participant then received a probabilistic hint about the other participant’s action ($80\%$ accurate) on the social-observation screen (blue dot). Finally, only after the partial observation of the other’s action did the participant receive a payout in the outcome-screen (following OL/OR action choice) or a probabilistic hint (following L action choice) about the tiger’s location, in the physical-observation screen (yellow dot). A “tiger trial” ended when either participant opened a door. Prediction, choice, social-observation, and physical-observation/outcome (all 5*sec*. duration) screens were all separated by the inter-event-interval ($1 sec. + \sim 600ms.$).

#### Differences in the original and modified TT

We took our original payout structure from the Doshi and Gmytrasiewicz formulation of the multiagent Tiger Problem, and their solutions using POMDP and I-POMDP frameworks^22,27^. In the single-agent version, an L action costs the participant $-1$ point (Fig. 4). This is comparatively a small price to pay to gain evidence that increases one’s likelihood of getting the reward of the gold pot and $+10$ points and of avoiding the tiger and losing $-100$ points. The original payout structure of the multiagent version of the task is structured such that the participants get their reward plus or minus half the reward of the other in the cooperative and competitive context respectively.

Verbal and written reports from participants completing the ITT with the original payout structure indicated a demotivating effect of opening the wrong door. That is, the losses were so large that some participants thought they could never make up for one loss with just such small gains for opening the correct door on subsequent rounds. Therefore, we modified the payout structure by doubling gains and halving losses for the door openings (Fig. 4). Doubling the gain ($+20$ points), and halving the losses ($-50$ points) decreased the severity of the punishment and the loss-to-gain ratio, and thus kept the participants more engaged. The payouts of the ITT also changed accordingly (see Payouts section below), and we used whole numbers to make it easier for the participants to keep track of rewards and reward totals.

Besides the differences in the payouts, in the modified TT, the participants were trained in the single-agent version of the task for 10 trials, and on the ITT (cooperative or competitive, depending on random assignment) for 20 trials.

### Payouts

We will describe the payout structure of the modified TT here. For a comparison between the original and the modified payout structures of the TT please refer to the supplement figure 1. The payout structure in Fig. 4 is for the scenario where the tiger is behind the left door; Changing the rows and columns of OL and OR would represent the tiger behind the right door. For the sake of convenience, we will call the two participants X and Y. We will refer to a particular cell in the payout matrix by its row and column number; As an example, row 1 column 2 would be {1,2}.

Payout in the single-agent version is straightforward, where the listen action costs the participant a single point {1,1}. When the participant opens the correct door {1,3} the reward is +20 points while encountering the tiger {1,2} incurs a loss of -50 points.

Payouts in the modified matrix in the ITT departed from the original matrix (see Fig. 4) and the structure of the payouts defined the interactional context . The modified payouts in the cooperative context of the multiagent version are completely symmetric to foster cooperation via shared outcomes. When the participants gather evidence through a listen action {1,1}, they lose $-1$ points each. The optimum scenario is when both open the correct door on the same round {3,3}, gaining the maximum $+20$ each. Other scenarios are relatively sub-optimal and incur losses. These moderate losses motivate participants to learn and look for the right door in subsequent trials. The worst scenario is when both participants get the tiger, losing $-50$ points each.

The main diagonal in the competitive context payout structure is similar to the cooperative one. Gathering evidence by listening costs each $-1$ points. The best-case scenario for X is to open the correct door when Y opens the wrong door {2,3}. This results in a large win ($+45$ points) for X, along with an even larger loss ($-60$ points) for Y. The payout structure also incentivizes a participant if the other is wrong, fostering competition to be the first one to the correct door.

### Indices of behavioral performance

Our goal in this paper is to characterize participants’ performance on the TT and on the ITT in two interactive contexts, cooperation and competition. We do this in terms of assessing the sequences and proportions of participants’ choices, along with their predictions about the actions of the other participant. We also aim to understand how other-regarding predictions relate to an agent’s choices by correlating the prediction and choice data. Finally, we also want to reveal learning-related changes and the ensuing improvement in task performance as both participants in the ITT learn about each other’s choices and make better predictions.

We derived several behavioral indices from the choice and the prediction data of the ITT. In this section, we explain how they relate to the interactive contexts and what ToM processes they suggest.

#### Choice indices

Here we will describe the different choice indices used to understand the behavior.

**Number of listen actions**

One important characteristic of choices in both the TT, but more so in the ITT, is the degree to which human agents seek information prior to acting. We operationalize this via the number of listen actions prior to an open action, which concludes a tiger-trial. In this respect, the number of listen actions can be interpreted as a data-driven index weighing uncertainties of reward with those of failure/loss. The number of listen actions should follow the incentivizing structure of the payout matrices in the different interactive contexts. Using I-POMDP modeling with the multiagent Tiger Problem^27,36^ allows us to determine optimal numbers of L actions depending upon two key parameters of I-POMDP models: the ToM level and the planning horizon (see work by Doshi et al.^36^ for complete modeling details and definitions of level and planning horizon). In brief, ToM “level” (see Rusch et al.^25^) is the level of recursion of Agent X’s model of Agent Y’s beliefs, intentions, values, etc. Level 0 ToM is defined as no ToM at all. Level 1 ToM is defined as basic ToM with no recursion, such that Agent X does have a model of Agent Y’s beliefs, intentions, values, but has no model of Agent Y’s model of Agent X. Level 2 ToM is defined as a recursive model, in which Agent X has a model of Agent X’s beliefs, intentions, values along with a model of Agent Y’s model of Agent X’s beliefs, intentions, values, etc. Levels of ToM above 2 are higher levels of recursion.

The planning horizon in such models refers to number of iterations from the current round whose estimated outcome probabilities influence the choice of action. Horizon 1 means that the model takes account only of the current round’s expectation. Horizon 2 means that the model looks one iteration ahead, and so on. We apply previously established models to provide optimal numbers of L actions for both the “Level 1, Horizon 1” (L1H1) and “Level 1, Horizon 2” (L1H2) models.

**Evidence difference**

Computational modeling reveals that several L actions are needed to gradually build up a belief about the tiger’s location because the physical observations in the TT (tiger growls) are only partial observations of the tiger’s true location (i.e., they are only $70\%$ accurate). Thus, the average evidence regarding the tiger’s location prior to an agent’s open action operationalizes that agent’s evidence threshold and allows an estimation of deviation from computationally optimal evidence accumulation. We calculate the evidence difference as the number of observations from the true location of the tiger minus the number of observations from the other side (e.g. if the tiger is on the left (TL), the evidence difference is calculated as $\mathrm {(nGL|TL)}-\mathrm {(nGR|TL)}$, where *n* stands for number and | for conditionality). Compared to the number of Listen actions the evidence difference effectively controls for the inconsistency in an observation sequence that arises from the probabilistic nature of the physical observations. We calculate the evidence difference for every tiger-trial and average them to obtain the subject-specific evidence threshold before an open action is committed.

**Identical open actions**

This measure provides the most direct link to the incentivizing structure of the payout matrices for the interactive contexts. During cooperation, the best outcome is to open the correct door together and the worst outcome is to open the tiger’s door together. During competition though, it is best to reach the correct door first, and even better if the other person chooses the wrong door on that round. So competition disincentivizes identical open actions. Because they are such a direct expression of contextual differences in the ITT, identical open actions lend themselves particularly well for demonstrating learning-related improvements of joint performance on the ITT.

**Correct open actions**

Wins or losses are the results of correct and incorrect open actions in the TT and ITT. The number of correct open actions is therefore a measure of how well human agents have understood and carried out the task, along with the quality of evidence on which those agents base their actions.

#### Prediction indices

While the above-mentioned measures characterize aspects of participant choice, indices calculated from prediction data on the ITT provide indirect assessments of ToM processes. A requirement for the most successful task performance in the ITT is that agents build mental models of other participants, which they can query implicitly or explicitly to best predict the other participant’s next action. To partially access and assess these representations, we used several behavioral indices from the prediction data.

**Number of listen predictions**

Paralleling the number of listen actions, the number of *predicted* listen actions or the number of listen predictions indicates how much evidence a participant thinks the other participant needs before committing an open action. Just as with the number of listen actions above, the number of listen predictions can be interpreted as the expected risk sensitivity of the other participant. Similarly, if the number of listen actions is expected to be higher during cooperation, the same holds for the number of listen predictions. For example, cooperatively playing participants with a higher need for evidence would expect the same from their co-participants, because they know that their co-participants are also aiming to coordinate the same actions for both participants.

**Prediction accuracy**

This is most direct behavioral measure of the veracity of a participants’ mental model of the other participant: if one participant can accurately predict the other participant’s actions, then mentalizing and/or action expectation representation are clearly successful. Prediction accuracy can be also seen as an expression of a successful Level 1 or higher ToM agent^25^. Such an agent is capable of representing what the other participant believes about the tiger’s location and the other participant’s expected values for actions.

**Logically consistent actions**

We define logically consistent actions as those participant choices that logically follow their predictions. For example, if a participant in a cooperative ITT believes that the other participant believes that the tiger is behind the left door and that therefore the other participant will open the door on the right in the current round, the participant will predict open-right. If the participant also believes that the tiger is behind the left door, then the participant should also open the right door on this round in order to maximize the probability of greatest reward. Of course, there will be rounds when a participant believes that the other participant is in fact wrong about the location of the tiger, but such trials are expected to be infrequent. In the context of a cooperative ITT, where same action coordination is beneficial for both participants, consistent actions are an important behavioral strategy for successful performance. If the participant predicts that the other participant will choose a specific action, then they would increases their own predictability, if they choose an action that is consistent with their own predictions. Therefore, consistent actions are a coarse indicator of Level 2 or higher ToM reasoning. Participants in the cooperative ITT know that they need to coordinate same actions, so it is beneficial for them to increase the predictability of their own actions. That is, assuming they are Level 2, they would choose a strategy that maximizes the chances that they can be predicted by a Level 1 ToM agent. Of course, while such sophistication of ToM reasoning is especially beneficial in the cooperative ITT (see also Discussion), Level 2 ToM could also benefit participants in the competitive ITT by allowing participants to act in less predictable ways based on their representations of other participants’ Level 1 ToM.

**Correctly predicted consistent actions**

Consistent actions are predicated on a participant’s predictions of the other participant and these predictions can be wrong. Therefore, we also look at a subset of consistent actions, namely those in which the predictions of the other participant were correct.

#### Other behavioral indices

Thinking about the other agent’s potential choices and incorporating these predictions into one’s own action selection process is cognitively demanding and requires considerable cognitive resources. We therefore expect an increase in reaction time (RT), whenever participants think about the other participant in detail. An important aspect of repeated social interaction in the ITT is that the participants can learn the other’s decision strategies and choice preferences. We look at the learning-related changes that this index brings to the ITT behavior.

In a final step, we wanted to link the prediction performance of the participant to their own choices and evaluate whether successful predictions of the other participant also resulted in better choices. We, therefore, provide several correlation analyses in which we link our prediction measures with several of the choice indices from above.

### Models of optimal performance

We aim to characterize the performance of our participants with respect to the performance of an optimal agent on the TT and the ITT. The computational model for the TT is a partially observable Markov decision process (POMDP), which was introduced by Kaelbling et al.^22^. It is a generic framework for decision-making under uncertainty, when agents do not have direct knowledge to states of the world, but only access those states through probabilistic observations. To accommodate such a decision-making scenario in an interactive context, Gmytrasiewicz et al.^26^ developed interactive POMDPs (I-POMDPs), a generalization of POMDPs to multiple interacting agent, which allows for the modeling of the other agents’ beliefs and actions within the computational model itself. In the following, we briefly describe these two frameworks and how they can solve the TT and the ITT. For more details (including the equations defining the model) we refer the interested read to the original publications^22,26^.

**POMDPs as a model for the single agent Tiger Task**

In a POMDP an agent does not have direct knowledge of the state of the world. In the case of the TT the two possible states of the world are “Tiger Left” and “Tiger Right” indicating the door, which hides the tiger that should be avoided. However, each state emits probabilistic observations, which are presented to the agent. In the case of the TT, these are the physical observations of “Growl Left” or “Growl Right” indicating the location of the tiger with an accuracy of $70\%$. Using these observations in a Bayesian belief updating scheme, the agent forms beliefs about the world in form of a probability distribution over states specifying the probability of each state in the current trial. These beliefs, which are updated in a so-called *state estimator*, are the basis for the decision of the agent, which action to take next.

More formally, a POMDP is defined by:

- a set of state S, which define the environment
- a set of action A, which an agent can take in each state
- a state transition function T: S$\times$A detailing the (possibly probabilistic) transitions between state of the environment conditional on the specific action
- a reward function R: S$\times$A detailing the immediate reward that an agent obtains when selecting action a in state s
- an observation function O: S$\times$A detailing the probability distribution of observations which the agent can make after performing action a in state s

The model performs Bayesian belief updating in the state estimator, which calculates the new beliefs of the agent based on the current state (unknown to the agent), the last action, and the observation that the agent has made. These updated beliefs (about the location of the Tiger) are the basis for the next action decision. These beliefs are filtered through quantal response equilibrium function (a sigmoid function) transforming beliefs into action probabilities, which are the model’s predictions for the next action by the agent.

We used a the POMDP implementation pomdp-solve by Toni Cassandra (code available at http://www.pomdp.org) to solve the TT using both the original and modified payout structure. The solver provides and optimal solution to POMDP in form of a policy graph that specifies the optimal action sequence given a specific observation and a particular belief. Specifically, we focused on the number of listen actions that the optimal agent would take (given the observation sequence of the participants in each trial) and compared this to the actual number of listen actions of the participants (averaging both over trials). In the case when the optimal agent reached an open action before the participant, we took the preceding number of listen action as the optimal number for this trial; in the case, when the participant committed an open action before the optimal agent, we simulated 30 additional observation sequences using the observation probabilities defined in the TT and recorded the number of listen actions (averaging over these simulations).

**I-POMDPs as a model for the multiagent Interactive Tiger Task**

An interactive POMDP (I-POMDP) generalizes the POMDP framework to a multi-agent setting, in which two or more agent simultaneously take actions in an uncertain environment. This is an additional level of complexity, because now the action of the other player has to be taken in account when calculating the next action. To solve this problem in a optimal way, the I-POMDP creates a model of the other players, which calculates their beliefs and action probabilities and then uses these simulated beliefs to compute the agent’s optimal action. Thus, an I-POMDP defines an “model within a model” and is thus a computational vehicle for assessing mentalizing in a quantitative way.

More formally, an I-POMDP has the same defining elements as a POMDP except that the set of state S is replaced by a set of interactive states IS: S$\times$M, which is the interaction of the states with the possible models M of the other agent. These M models individually also contain the main agents’ models which recursively do the same and so on. When calculating the beliefs, the I-POMDP first calculates the possible beliefs of the other agent given the observations that the other agent make, and then marginalizes over the other agent’s beliefs to compute their own belief update. This is then filtered again through a quantal response equilibrium function to obtain action probabilities for the next decision. For more details on the I-POMDP and the implemented quantal response equilibrium function, please see^26,37^.

Including a model of the other agent in the belief update of the I-POMDP raises the question of the level of recursion of these “models within models”^38,39^. A Level 0 I-POMDP agent (essentially a POMDP agent) does not have a model of the other agent and learns only from the observed environment. A Level 1 I-POMDP agent constructs a model the other agent as a Level 0 agent, so the first agent thinks that the other agent does not have a model of the first agent. A Level 2 agent builds a model of the other agent as a Level 1 agent, which includes a model of the first agent as a Level 0 agent. In summary, a Level n I-POMDP agent constructs a model of the other agent at the Level n-1. The level of recursion in the I-POMDP affects how the I-POMDP agent calculates and anticipates the other agent’s actions.

Another important determinant of the belief update in the I-POMDP is the planning horizon. The value iteration algorithm that is used to solve the I-POMDP iterates over the number of planning steps while marginalizing over all possible own and other actions to calculate and update beliefs for the next decision. Especially for tasks like the ITT, in which there are several evidence-gathering steps, the valuation of actions, which are based on the beliefs, can be significantly affected by the number of steps the algorithm is allowed to look ahead while considering all possible joint actions.

For the comparison of the performance of our participants on the ITT with that of an optimal agent, we used a similar strategy to the single-agent TT. However, due to the high computational demand of the I-POMDP, an optimal solution using value iteration^40^ is commonly not possible. Hence, the optimal solution has to be approximated. We used an interactive particle filter implemented in C++^27,41^ to approximate optimal performance given the actual observation sequences that our participants experienced. We also limited the optimal I-POMDP agent to be a Level 1 agent with a planning horizon of either 1 or 2 steps with 1000 particles.

As in the case of the single-agent TT, whenever the I-POMDP reached an open action before the participant in a particular trial we took this as the optimal number of listen actions for this trial. However, when the participant committed an open action before the I-POMDP, we simulated 30 possible observation sequences based on the observation probabilities of the ITT and recorded the number of listen action until the I-POMDP reached an open action. Then we averaged over simulations to obtain the optimal number of listen action for this trial. Finally, as in the case of the single-agent TT and the POMDP we averaged across all 30 tiger trials to obtain a subject-specific optimal number of listen actions.

### Statistical analyses

All statistical tests were performed in R version 4.0.3^42^ with the help of tidyr package^43^ and the figures are generated with the help of ggplot and supporting packages^44,45^. For comparisons of the means of the interactive contexts, we performed an independent Welch two-Sample t-test between the sample means and report the t-value, degrees of freedom, p-value, the 95% confidence interval and the effect size with Cohen’s d-value. We also corrected for multiple comparisons using Bonferroni corrections, setting the criteria for the significance at the value of $p< 0.006$ (0.05/8). For the comparison between the optimal POMDP model and the participant behavior we performed a two-way mixed effects ANOVA treating participant behavior and the optimum POMDP model as the within-subject factor and the two matrix versions as the between-subject factor. Similarly, another two-way mixed effects ANOVA treating participant behavior and the optimum I-POMDP model as within-subject factor, and the two contexts as the between-subject factor was performed for the multiagent ITT.

For the comparisons of learning effect through sessions between contexts, we performed two-way mixed effects ANOVAs treating session as a within-subject factor and context as a between-subject factor with significance set at $p<0.05$. From these analyses we report the F-statistic degree of freedom between and within, the F-ratio and the p-value. Differences in the correlation coefficients were tested at the significance level of $p<0.05$ using Fishers’ z-transformation by calculating the *z_observed* value.

$$\begin{aligned} z_\text {observed}= \frac{(z_1 - z_2)}{\sqrt{(1 / N_1 - 3) + (1 / N_2 - 3)}} \end{aligned}$$

Where, $z_1$ and $z_2$ are the Fisher z-transforms of the two correlation coefficients, while $N_1$ and $N_2$ are their respective sample sizes. Statistical significance at the 5% level is achieved when this value is to be beyond a critical threshold of $\pm 1.96$. A $z_\text {observed}$ beyond the critical threshold is an indicator of significance and the rejection of null hypothesis.

## Supplementary Information

Supplementary Information.

## Data Availability

The PIs on this project commit to sharing the data publicly on the National Science Foundation CRCNS site once the initial descriptive and IPOMDP modeling papers are accepted for publication.
