# Supplementary material for: Humans depart from optimal computational models of interactive decision-making during competition under partial information
Source: Sci Rep. 2022 Jan 7;12:289. doi: 10.1038/s41598-021-04272-x (PMC8741801; doi:10.1038/s41598-021-04272-x)
Supplement: Supplementary file 1 — Supplementary Information. [file 41598_2021_4272_MOESM1_ESM.pdf]

Supplement:

Humans depart from optimal computational models of interactive decision-making during competition under partial information

Saurabh Steixner-Kumar[1,*], Tessa Rusch[1,2], Prashant Doshi[3], Michael Spezio[4,1,+,*], and Jan Gläscher[1,+,*]

[1]Institute of Systems Neuroscience, University Medical Center Hamburg-Eppendorf, Germany
[2]Division of the Humanities and Social Sciences, California Institute of Technology, CA, USA
[3]Department of Computer Science, University of Georgia, GA, USA
[4]Psychology, Neuroscience, and Data Science, Scripps College, CA, USA
[*]correspondence: s.steixner-kumar@uke.de; mspezio@scrippscollege.edu; glaescher@uke.de
[+]these authors contributed equally to this work

Supplement figure

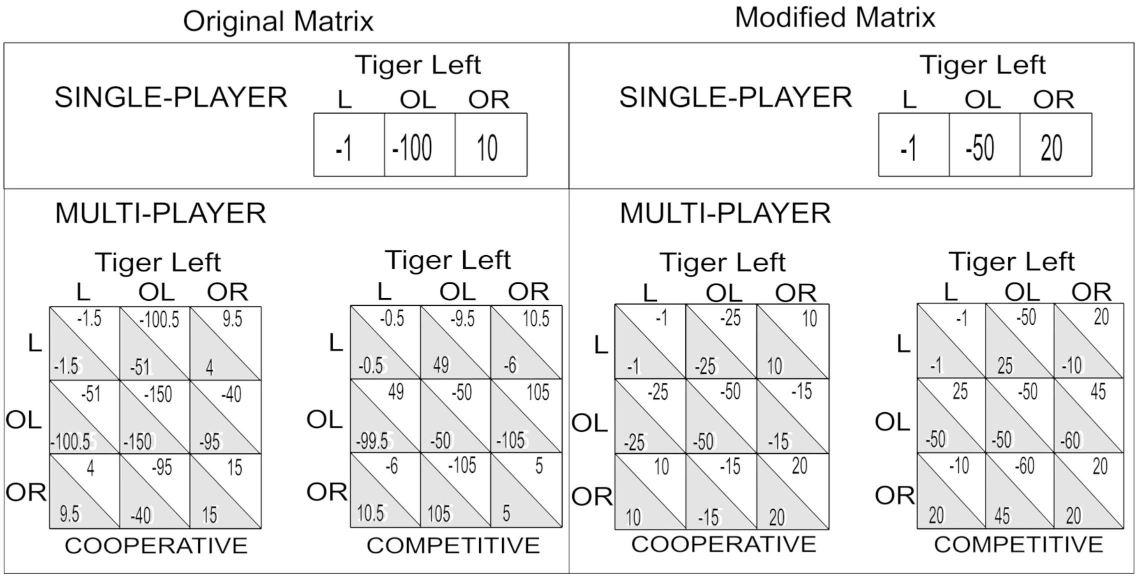

Figure 1. Original and modified payout structures of the Tiger Tasks. The payouts are potential points that can be gained for a chosen action (listen (L) and open-left/right (OL/OR)), when the tiger is behind the left door. The points scheme remains the same for the tiger behind the right door if we switch the OL and OR columns and rows. In the original matrix for the single-participant setting, a L action costs -1 points. Getting the gold-pot rewards +10 points while encountering the tiger takes away -100 points. In the multiagent setting, the column actions represent one's own actions, while the row is the other participants' actions. The point system is similar from the single-participant setting but more combinations are added as the other participants' actions affect the points a participant can make. Depending upon the context, the points a participant gains are their own points plus half the points of the other participant (cooperative context) or minus the points of the other participant (competitive context). In the modified matrix single-participant setting the gains are doubled to +20 points and the losses are halved to -50 points. The multiagent setting has simpler whole numbers and the differences in the points is comparatively less extreme. To facilitate more cooperation in the cooperative context, the payout in the modified matrix is completely symmetric.

Linear mixed-effects regression model comparison

Table s1 lists the different linear mixed-effects regression models used to explain the task performance of the participants and both IPOMDP models. The models include the number of Listen actions (nListen), the number of correct Open actions (CorrOpen) and the number of identical Open actions (IdentOpen) of both agents in the dyad, and the 'group' variable that codes for participants, L1H1, and L1H2 models. As random effects we included a random intercept for each participant (or modeled subject in L1H1 and L1H2).

Model	Formula
M01	TotalScore ∼ 1 + group + nListen + (1 | SubjectID)
M02	TotalScore ∼ 1 + group + nListen + IdentOpen + CorrOpen + (1 | SubjectID)
M03	TotalScore ∼ 1 + group + nListen + IdentOpen + CorrOpen + (1 + nListen | SubjectID)
M04	TotalScore ∼ 1 + group + nListen + IdentOpen + CorrOpen + (1 + IdentOpen | SubjectID)
M05	TotalScore ∼ 1 + group + nListen + IdentOpen + CorrOpen + (1 + CorrOpen | SubjectID)
M06	TotalScore ∼ 1 + group + nListen + IdentOpen + CorrOpen + nListen:IdentOpen + (nListen | SubjectID)
M07	TotalScore ∼ 1 + group + nListen + IdentOpen + CorrOpen + nListen:IdentOpen + nListen:CorrOpen + (nListen | SubjectID)
M08	TotalScore ∼ 1 + group + nListen + IdentOpen + CorrOpen + nListen:IdentOpen + nListen:CorrOpen + IdentOpen:CorrOpen + (nListen | SubjectID)
M09	TotalScore ∼ 1 + group + nListen + IdentOpen + CorrOpen + nListen:IdentOpen + nListen:CorrOpen + IdentOpen:CorrOpen + nListen:IdentOpen:CorrOpen + (nListen | SubjectID)
M10	TotalScore ∼ 1 + group + nListen + IdentOpen + CorrOpen + nListen:IdentOpen + nListen:CorrOpen + IdentOpen:CorrOpen + nListen:IdentOpen:CorrOpen + (1 | SubjectID)
M11	TotalScore ∼ 1 + group + nListen + IdentOpen + CorrOpen + nListen:group + nListen:IdentOpen + nListen:CorrOpen + IdentOpen:CorrOpen + nListen:IdentOpen:CorrOpen + (1 | SubjectID)
M12	TotalScore ∼ 1 + group + nListen + IdentOpen + CorrOpen + nListen:group + IdentOpen:group + CorrOpen:group + nListen:IdentOpen + nListen:CorrOpen + IdentOpen:CorrOpen + nListen:IdentOpen:CorrOpen + (1 | SubjectID)

Table s1. Linear mixed-effects regression models. Different models used to explain the task performance.

Comparing these models revealed that model M12 provided the best fit to the data in both contexts. Table s2 compares all the models while table s3 compares the estimates of the best fit model in both contexts.

	Competitive			Cooperative		
Model	AIC	BIC	Log Likelihood	AIC	BIC	Log Likelihood
M01	1781.63	1800.48	-884.82	1898.15	1917.88	-943.07
M02	1472.15	1497.29	-728.08	1673.24	1699.55	-828.62
M03	1475.97	1507.38	-727.98	1649.28	1682.16	-814.64
M04	1447.02	1478.44	-713.51	1643.58	1676.46	-811.79
M05	1435.87	1467.29	-707.94	1664.56	1697.44	-822.28
M06	1472.33	1506.89	-725.17	1644.93	1681.10	-811.47
M07	1467.80	1505.50	-721.90	1606.70	1646.16	-791.35
M08	1346.06	1462.35	-697.75	1587.83	1630.57	-780.91
M09	1413.48	1457.47	-692.74	1580.42	1626.46	-776.21
M10	1410.84	1448.54	-693.42	1607.01	1646.46	-791.50
M11	1395.90	1439.89	-683.95	1595.91	1641.95	-783.96
M12	1346.06	1402.61	-655.03	1554.40	1613.59	-759.20

Table s2. Statistical linear mixed-effects regression model comparison (both context).

	Competitive	Cooperative
	estimate (standard error)	estimate (standard error)
(Intercept)	$-371.53^{***}(92.46)$	$-140.57^{*}(58.60)$
group1	$-73.06^{*}(33.01)$	$-31.16(26.22)$
group2	$-8.67(45.77)$	$27.66(24.04)$
nListen	$31.79(22.04)$	$8.10(11.61)$
IdentOpen	$48.67(189.04)$	$-347.54^{*}(173.33)$
CorrOpen	$463.71^{***}(121.41)$	$205.24^{**}(77.83)$
group1:nListen	$-1.68(7.98)$	$2.40(6.97)$
group2:nListen	$7.34(4.98)$	$-4.38(4.47)$
group1:IdentOpen	$-42.68(21.85)$	$-5.11(12.85)$
group2:IdentOpen	$77.04^{**}(27.83)$	$-33.64^{***}(9.95)$
group1:CorrOpen	$122.86^{***}(36.29)$	$35.72(29.95)$
group2:CorrOpen	$-69.74(56.22)$	$-9.99(27.72)$
nListen:IdentOpen	$-93.37^{*}(45.95)$	$3.92(44.04)$
nListen:CorrOpen	$-31.09(26.56)$	$-14.11(14.86)$
IdentOpen:CorrOpen	$85.01(234.19)$	$421.75(215.37)$
nListen:IdentOpen:CorrOpen	$69.53(50.85)$	$0.33(52.73)$
AIC	1346.06	1554.40
BIC	1402.61	1613.59
Log Likelihood	-655.03	-759.20
Num. obs.	171	198
Num. groups & SubjectID	57	66
Var & SubjectID (Intercept)	67.27	96.53
Var & Residual	189.55	149.39

$^{***}p < 0.001$; $^{**}p < 0.01$; $^{*}p < 0.05$

Table s3. Linear mixed-effects regression model estimates comparison. The best models in both interactive context is summerized in this table. The group1 variable represents IPOMDP L1H1 model and group2 variable represents IPOMDP L1H2 model, with the participants as baseline.
